# Postdiagenetic Changes in Kerogen Properties and Type by Bacterial Oxidation and Dehydrogenation

**DOI:** 10.3390/molecules27082408

**Published:** 2022-04-08

**Authors:** Agnieszka Wilamowska, Marta Koblowska, Renata Matlakowska

**Affiliations:** 1 Department of Geomicrobiology, Institute of Microbiology, Faculty of Biology, University of Warsaw, Miecznikowa 1, 02-096 Warsaw, Poland; awilamowska@iimcb.gov.pl; 2 International Institute of Molecular and Cell Biology in Warsaw, Księcia Trójdena 4, 02-109 Warsaw, Poland; 3 Laboratory of Systems Biology, Faculty of Biology, University of Warsaw, Miecznikowa 1, 02-096 Warsaw, Poland; mk.koblowska@uw.edu.pl; 4 Institute of Biochemistry and Biophysics, Polish Academy of Sciences, Pawińskiego 5A, 02-106 Warsaw, Poland

**Keywords:** kerogen, shale rock, Rock-Eval, bacterial community, oxygenation, dehydrogenation

## Abstract

A significant part of organic carbon found on the earth is deposited as fossil organic matter in the lithosphere. The most important reservoir of carbon is shale rocks enriched with organic matter in the form of kerogen created during diagenesis. The purpose of this study was to analyze whether the bacterial communities currently inhabiting the shale rocks have had any impact on the properties and type of kerogen. We used the shale rock located on the Fore-Sudetic Monocline, which is characterized by oil-prone kerogen type II. We were able to show that shale rock inhabited by bacterial communities are characterized by oxidized and dehydrated kerogen type III (gas-prone) and type IV (nonproductive, residual, and hydrogen-free). Bacterial communities inhabiting shale rock were dominated by heterotrophs of the *Proteobacteria*, *Firmicutes*, and *Actinobacteria* phyla. Additionally, we detected a number of protein sequences in the metaproteomes of bacterial communities matched with enzymes involved in the oxidative metabolism of aliphatic and aromatic hydrocarbons, which may potentially contribute to the postdiagenetic oxidation and dehydrogenation of kerogen. The kerogen transformation contributes to the mobilization of fossil carbon in the form of extractable bitumen dominated by oxidized organic compounds.

## 1. Introduction

Kerogen is a polycondensed macromolecular fossil organic matter deposited in sedimentary rocks. It is the most abundant form of organic carbon found on the earth, and its total mass corresponds to greater than 10^15^ tons of carbon [1]. Kerogen is insoluble in organic solvents and inorganic nonoxidizing acids. This property of kerogen results from its macrostructure, which is characterized by a unique composition consisting of various interrelated organic compounds of high molecular weight. The main components of kerogen are long-chain aliphatic hydrocarbons (HCs) and polycyclic aromatic HCs.

The chemical and physical properties of kerogen are determined by the type of its precursor molecules, the conditions of sedimentation, and the diagenetic transformations of the molecules over millions of years [2]. Based on the scheme of changes in the elemental composition (H/C-to-O/C ratio) during the thermal maturation of various carbon macerals, four basic types of kerogen have been distinguished [3]. Kerogen type I forms in shallow lakes and lagoons that are depleted of oxygen and inhabited by freshwater algae [2]. During the process of thermal maturation, this type of kerogen generates liquid HCs (oil). Kerogen type II is usually formed under reducing conditions in the marine environment, where phytoplankton acts as the main source of organic matter. This type of kerogen is highly capable of generating liquid and volatile HCs (oil and natural gas) [2]. Kerogen type III forms from vascular plants in terrestrial environments and can generate volatile HCs (natural gas) [2]. The rarest is kerogen type IV and mainly contains heavily transformed and oxidized organic debris of various origins. Its macrostructure consists mainly of polycyclic aromatic compounds and is characterized by a very low H/C ratio (<0.5) and a high O/C ratio (0.02–0.30) resulting from oxidation. This type of kerogen has very low or no potential to generate HCs [2].

The purpose of our study was to investigate whether the bacteria currently inhabiting the shale rocks have any impact on the properties and type of kerogen. The shale rock located on the Fore-Sudetic Monocline (SW Poland) (Supplementary Information A, Figure S1) was used for the study. This shale rock was formed in the shallow Zechstein Sea around 256 million years ago during the Permian period (Lopingian). The rock is deposited at a depth of 380–1300 m below the earth’s surface, with a thickness ranging from several centimeters to almost 2 m (average 30–60 cm). The fossil organic matter present in the shale rock is of marine origin and consists of a small admixture of land-borne material [4]. The content of this organic matter can reach up to 30 wt% (average 6 wt%). It occurs in the form of type II kerogen, constituting ~95–99 wt% of organic carbon [4,5]. The rest of the organic matter (~1–5 wt%) is a solid bitumen extractable by organic solvents [5].

In this study, we used the samples of shale rock taken from profiles exposed during mining operations in underground copper mines. For testing, we selected six research stands in different locations of the mines but similar physicochemical conditions. We collected three samples 2–3 m apart at each research site. The first series of samples (stands 1 and 2) were collected from profiles created a few days before sampling, while the rest of the samples (stands 3–6) came from old profiles that were estimated to be over 10 years old (the exact timing of the shale rock exposure had not been documented in the past). Characteristics of research stands are presented in Supplementary Information A (Table S1).

In our research, we hypothesized that shale rock exposed as a result of mining activity (stands 3–6) underwent chemical and biological weathering processes and was exposed to the activity of water and air, but it was also inhabited by microorganisms, both indigenous and allochthonous, introduced into the mine’s environment by water, as well as ventilation systems. This action could proceed in different ways depending on the physical and chemical conditions prevailing in a given stand (temperature, humidity, oxygen content). Summarizing the above hypothesis, we assumed that shale rock collected from sites 3–6 was subjected to bio/weathering processes for a minimum of 10 years while the solid shale rock from stands 1 and 2 (exposed a few days before sampling) constitute a type of control and represent the most characteristic kerogen for the studied shale rock deposit, i.e., according to the literature, it is kerogen II. Our research included kerogen type determination of all collected samples and subjected them to microbiological analyses. From these analyses, we detected bacteria and checked their viability. Then, we analyzed the taxonomic composition of bacteria and the bacterial metaproteomes to identify the enzymes which potentially may affect the kerogen.

## 2. Results and Discussion

### 2.1. Properties and Type of Kerogen in Shale Rock on the Fore-Sudetic Monocline

We subjected all the collected samples to pyrolytic analysis using the Rock-Eval method. This method is used for the analysis of reservoir geochemistry and allows the degree of maturity of kerogen to be estimated as well as its genetic type and ability to generate volatile and solid HCs [6]. The key kerogen parameters tested in the analysis were the hydrogen index (HI) reflecting the number of HCs generated from 1 g of total organic carbon (TOC) (mg HCs/g TOC), and the oxygen index (OI), which is the amount of CO_2_ generated from 1 g of TOC (mg CO_2_/g TOC), indicating the degree of kerogen oxidation. The other parameters tested were the content of pyrolytic carbon (PC), residual carbon (RC), and free hydrocarbons (FHCs), hydrocarbon potential (HCP), and maturity temperature (T_max_). Based on the estimated values of these parameters, the type of kerogen was determined. Next, we selected shale rock samples, which differed in the abovementioned parameters, and subjected them to microbiological analyses.

**Figure 1:**
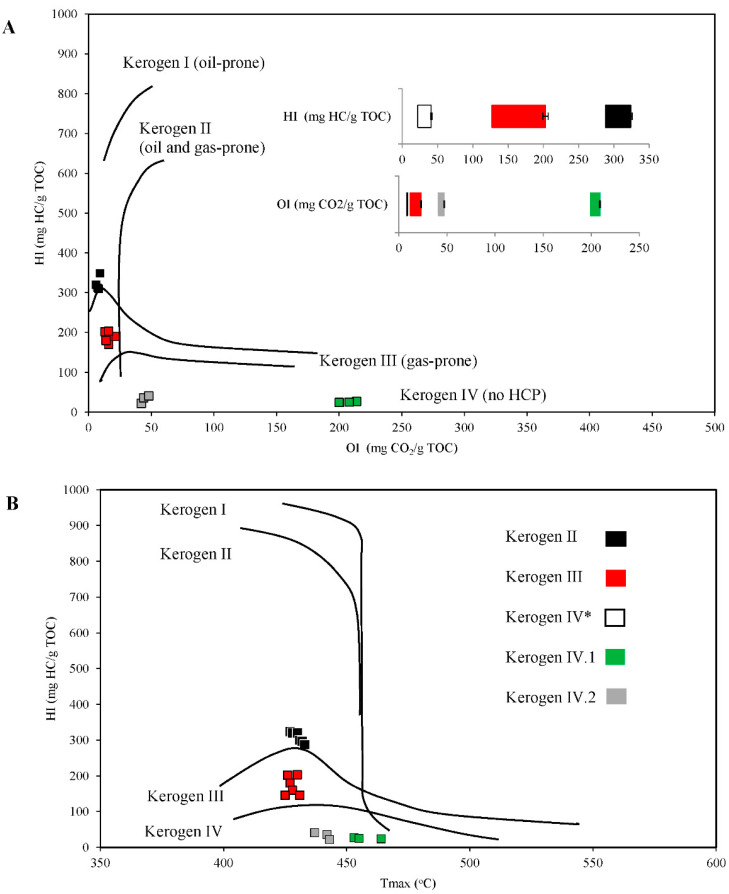

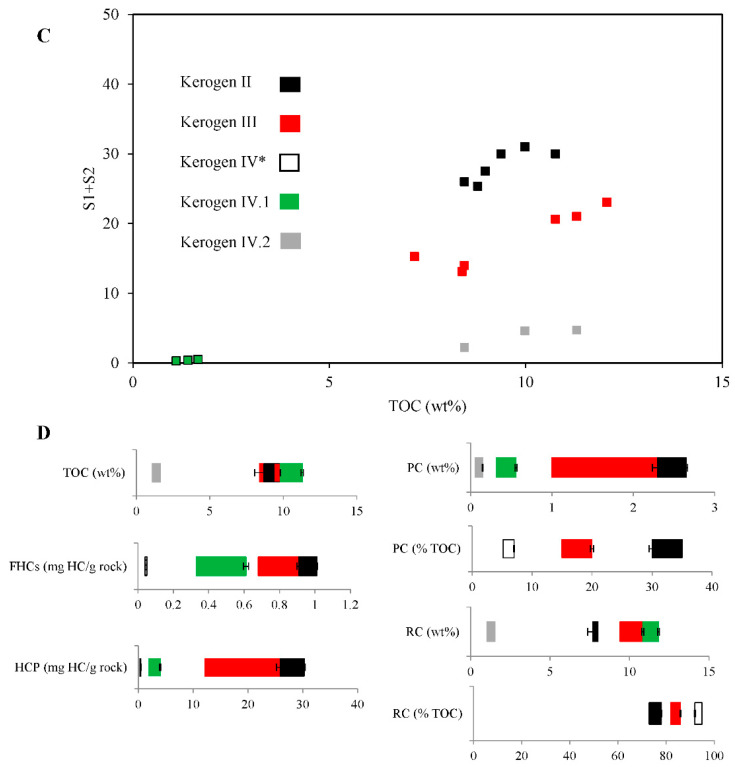
Pyrolytic characteristics of shale rock subjected to the Rock-Eval analysis. (**A**) Modified Van Krevelen diagram showing the correlation of hydrogen (HI) and oxygen index (OI). (**B**) HI and maturity temperature (T_max_) crossed diagram. (**C**) Correlation of total organic carbon (TOC) and hydrocarbon potential (S1 + S2). (**D**) Ranges of pyrolytic parameters of kerogen (FHCs—free hydrocarbons; HCP—hydrocarbon potential; PC—pyrolytic carbon; RC—residual carbon). (*) Parameters whose values are similar for type IV.1 and IV.2 kerogen samples are presented together (white square), while those that differ significantly are presented separately (green and gray squares). All the differences are statistically significant (significance level: *p* < 0.05). The error bars represent standard deviation.

Although the shale rock on the Fore-Sudetic Monocline is described in the literature as containing kerogen II organic matter [5], our research based on the Rock-Eval pyrolytic analysis revealed that there were three types of kerogen among the tested shale rock samples: type II, III, and IV. The results of this analysis are shown in Figure 1A–D.

The first series of samples taken from stands 1 and 2 (the profile created a few days before sampling) were characterized by type II kerogen. The TOC content of the samples was 8.78–9.37 wt%, and their composition was 72–75% RC and 25–28% PC. The content of FHCs was 0.91–1.01 mg HCs/g rock, while HCP equal to the number of HCs formed during kerogen pyrolysis ranged from 25.26 to 30.38 mg HCs/g rock. The thermal maturity degree of the organic matter present in the tested rock samples, corresponding to the maximum pyrolysis temperature, was in the range of 427–432 °C. The HI of the samples was estimated at 288–324 mg HCs/g TOC, while the OI was approximately 8–9 mg CO_2_/g TOC. The obtained results prove that the tested samples were potentially excellent parent rocks in terms of HC generation which have not fully realized their original HCP. The thermal maturity degree of these samples was below 435 °C, which is typical of immature rocks [2]. The results obtained indicate that the organic matter contained in these samples was of an oil-prone type.

The second series of shale rock samples taken from stands 3 and 4 (the profiles created > 10 years before sampling) were identified as kerogen type III and had a TOC content of 7.17–13.74 wt%. Their composition was 82–86% RC and 15–20% PC. The content of FHCs in these samples was 0.67–1.52 mg HCs/g rock, while the HCP ranged from 12.27 to 26.01 mg HCs/g rock. The degree of thermal maturity of these samples was determined to be approximately 425–430 °C. The HI ranged between 127 and 203 mg HCs/g TOC, while the OI was in the range of 12–23 mg CO_2_/g TOC. To sum up, the organic matter contained in this set of shale rock samples was of a gas-forming type.

The third series of shale rock samples taken from stands 5 and 6 (the profiles created > 10 years before sampling) were identified as kerogen type IV and were characterized by completely different parameters and also showed significant differences among themselves, primarily in the content of TOC and the degree of oxidation (OI). The results indicate that this type of kerogen can be distinguished into two subtypes, which we marked: kerogen IV.1 and kerogen IV.2. In Figure 1, the pyrolytic analysis parameters that were similar for these two samples (HI, PC (%), and RC (%)) are shown together, whereas those that were significantly different (TOC, OI, FHCs, HCP, PC (wt%), and RC (wt%)) are shown separately.

The samples of shale rock identified as kerogen type IV.1 had a very low content of TOC (1.1–1.66 wt%). Their content of FHCs was also very low, ranging from 0.04 to 0.67 mg HCs/g rock, while their HCP values ranged from 0.26 to 0.45 mg HCs/g rock. The degree of thermal maturity of these samples was 453–464 °C. The HI ranged between 24 and 27 mg HCs/g TOC, while the OI was very high and ranged between 200 and 210 mg CO_2_/g TOC.

The samples of shale rock identified as kerogen type IV.2 had a similar HI (22–41 mg HCs/g TOC) as kerogen IV.1, but a much lower OI (42–48 mg CO_2_/g TOC). Their TOC content was higher and reached 8.44–11.3 wt%. The content of FHCs in these samples was in the range of 0.33–0.6 mg HCs/g rock, while the HCP ranged from 1.88 to 4.09 mg HCs/g rock. The degree of thermal maturity of these samples was estimated to be about 437–443 °C. However, both samples representing kerogen type IV comprised 92–95% RC and 2–7.7% PC.

Summing up, the organic matter contained in these two types of shale rock samples was degraded and hydrogen-depleted, but in the case of type IV.1, the organic matter was strongly oxidized. Kerogen type IV is usually considered as a product obtained by strong chemical and biological degradation of kerogen type III or II, and thus cannot be treated as a significant source of HCs [2].

**Figure 2:**
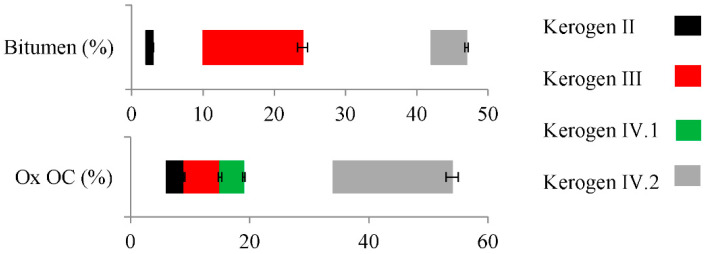
The content of bitumen and the content of oxidized organic compounds (Ox OC) in kerogen II, III, IV.1, and IV.2 samples. All the differences are statistically significant (significance level: *p* < 0.05). The error bars represent standard deviation.

In addition, an interesting feature observed in the studied shale rock samples was the differences in the content of extractable organic matter (referred to as bitumen) and oxidized compounds (Figure 2). In the kerogen type III samples, the bitumen content was 10–24%, and 42–47% in kerogen type IV samples, while in kerogen type II samples, the content was only 2–3%. This result may indicate the mobilization of simpler organic compounds from kerogen to the extractable fraction. The content of the oxidized organic compounds (kerogen II: 6–9%, kerogen III: 9–15%, kerogen IV.1: 15–19%, kerogen IV.2: 33–55%) was in agreement with OI, suggesting the oxidative nature of the transformation processes.

Thus, the results of the pyrolytic analysis show that the kerogen found in the shale rock samples of Fore-Sudetic Monocline is very diverse, even though these rocks arose under the same conditions—from the potentially good parent rocks in terms of HC generation, which had not fully realized their original HCP (type II kerogen), to rocks with gas-forming type III kerogen, and finally to the parentless rock in terms of HC generation containing degraded, hydrogen-depleted, and/or oxidized type IV kerogen.

When interpreting the results obtained, it is important to note that the weathering processes of kerogen are extremely difficult to study due to the complexity of its structure, as well as the significant heterogeneity of shale rock and also the possibly changing physical and chemical environmental conditions during bio/weathering. Several reports have documented the oxidative paleoweathering of fossil organic matter present in shale rocks [7,8,9,10,11]. They have shown that weathering results in selective leaching of macromolecules from kerogen and alters the concentration and molecular composition of the extractable organic compounds. Studies analyzing this process in detail have revealed significant changes in the distribution of aliphatic and aromatic HCs, including the polycyclic compounds. Their results suggest that the transformation of HCs plays a crucial role in the weathering of fossil organic matter. However, the impact of paleoweathering processes on types of kerogen has so far not been investigated.

Furthermore, little is known about the importance of bacteria in the weathering processes of kerogen. Thus far, only a few reports have shown the role of microorganisms in the degradation of terrestrial black shale organic matter in the field [12,13] and in laboratory conditions [14,15]. However, the changes caused in kerogen by microbial activity have not been analyzed in these studies. So far, only our earlier studies have analyzed the effect of bacteria on kerogen in laboratory conditions [16] and in the field [17]. In these studies, we described the oxidation and dehydrogenation of kerogen caused by the bacterial consortium. The laboratory study demonstrated that long-term (360 days) bacterial activity caused the alteration of kerogen II, which shifted closer to the parameters of kerogen III. These changes were not observed in the sterile controls, in which the black shale was only subjected to the action of water and oxygen in the study [16]. Similarly, the field studies performed by Włodarczyk et al. [17] on black shale showed the impact of lithobiontic microbial communities on the transformation of kerogen II into kerogen IV.

### 2.2. Detection of Microorganisms and Determination of Their Viability Based on Metabolic Activity

For simplicity, the sample series enriched with specific types of kerogen will be referred to as kerogen II, kerogen III, and kerogen IV (IV.1 and IV.2) in further parts of the manuscript.

**Figure 3:**
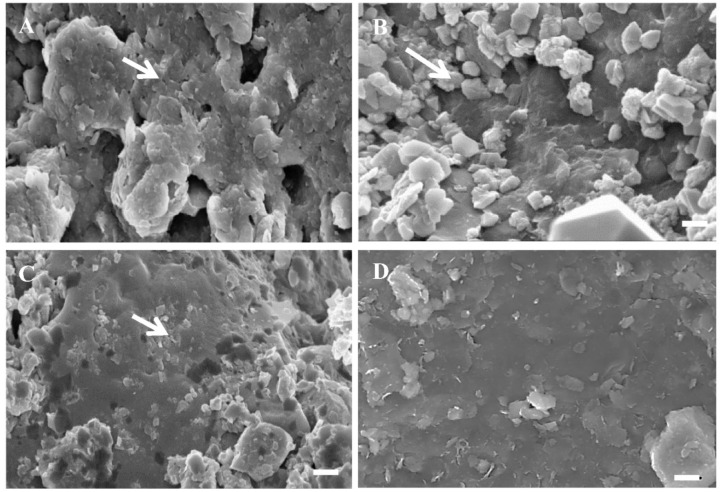
Bacterial biofilm on the surface of shale rock enriched in: (**A**) kerogen III, (**B**) kerogen IV.1, and (**C**) kerogen IV.2. (**D**) The surface of shale rock enriched in kerogen II. Arrows—cell-like structures detected on shales (SEM images), bar—2 µm.

The microbiological analyses showed that all the tested shale rock samples, which were characterized by type III and IV kerogen, were inhabited by microorganisms. Figure 3A–C shows the biofilm-like structures observed under the scanning electron microscope. No microorganisms were detected on kerogen II (stands 1 and 2) (Figure 3D).

**Figure 4:**
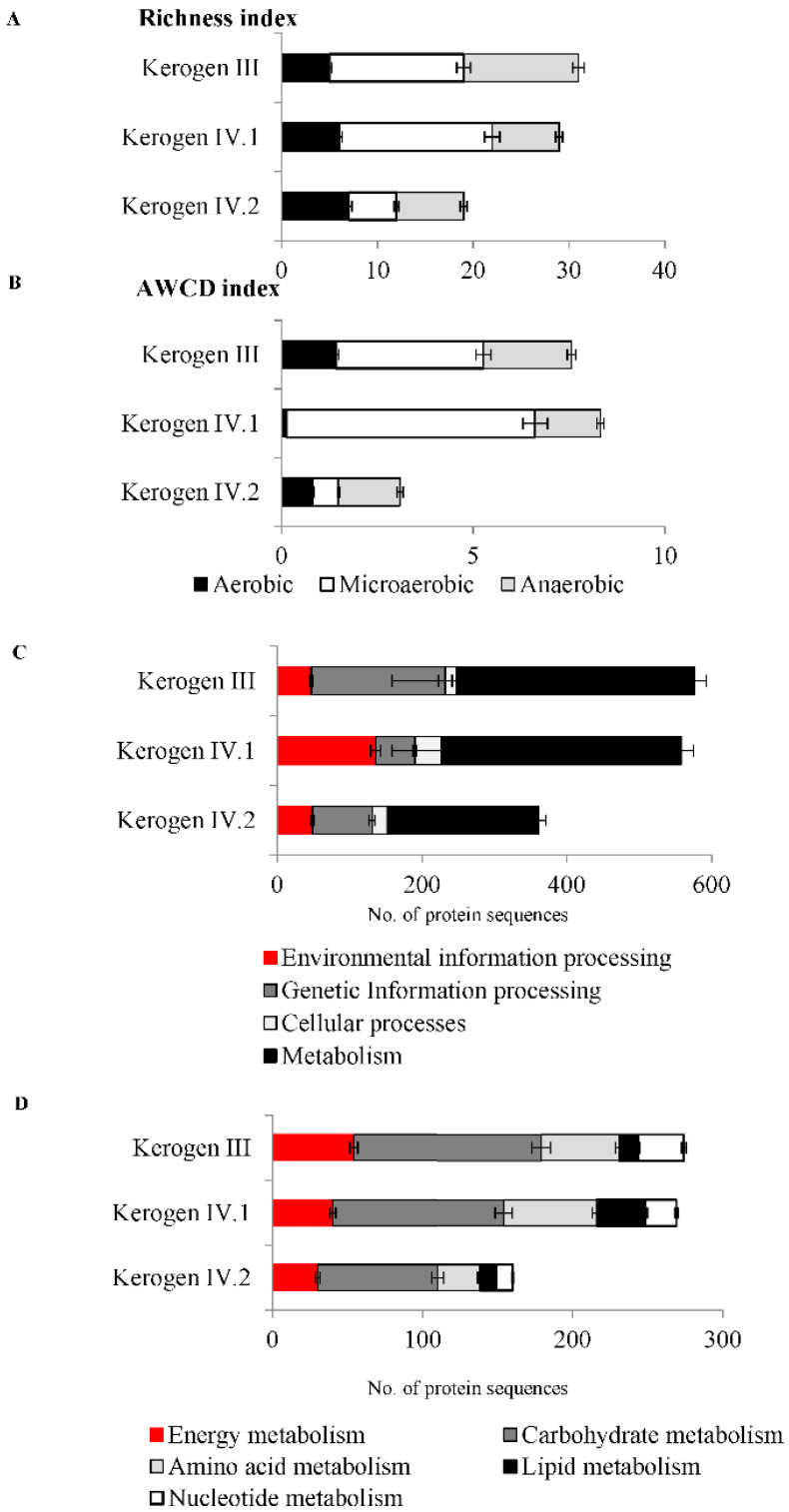
Activity and general characteristics of metaproteomes of the three bacterial communities identified in the shale rock samples enriched in kerogen III, IV.1, and IV.2 samples. Metabolism profile of organic carbon expressed as richness index (**A**) and average well color development (AWCD) index (**B**) under aerobic, microaerobic, and anaerobic conditions. Number of protein sequences detected in metaproteomes, classified into functional categories (**C**) and main categories of metabolism (**D**). The error bars represent standard deviation.

The microorganisms inhabiting the shale rock were observed to be metabolically active, capable of degrading selected organic compounds under aerobic, microaerophilic, and anaerobic conditions (Figure 4A,B and Supplementary Information A, Figures S2–S4). The results indicate that the microorganisms inhabiting kerogen III had the highest metabolic activity corresponding to the number of degraded organic compounds (richness index), while degradation intensity (average well color development—AWCD index) was the highest in the case of kerogen IV.1. The analyzed kerogen II sample did not show any microbial activity.

The mass-spectrometry-based analysis performed for protein identification revealed a total of 1735, 2297, and 810 sequences of bacterial proteins in the metaproteomes of the three bacterial communities inhabiting shale rock enriched in kerogen types III, IV.1, and IV.2, respectively (Supplementary Information B). More than one-third of these sequences were identified to be hypothetical proteins of unknown function. Figure 4C,D show the number of sequences matched with proteins detected in the metaproteomes of the three bacterial communities studied, then classified into four functional categories (Figure 4C) and main categories of metabolism (Figure 4D).

In conclusion, the results presented in Figure 3 and Figure 4 confirm the presence and the viability of bacteria on shale rock characterized by kerogen III and IV and show the overall characteristics of their metaproteome.

### 2.3. Bacterial Communities Inhabiting the Shale Rock

**Figure 5:**
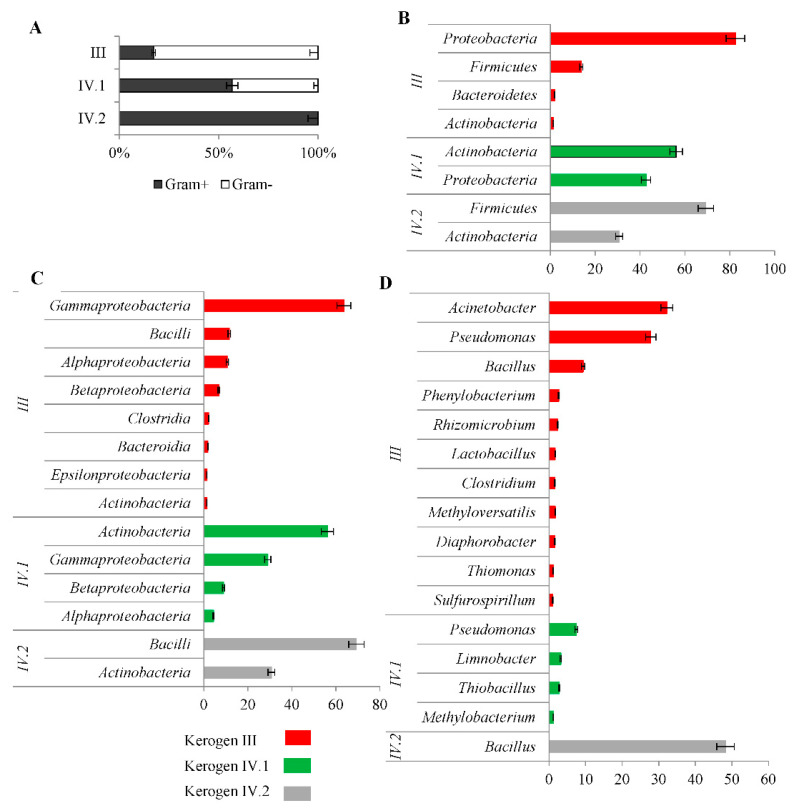
Taxonomic composition of the bacterial communities identified in the shale rock samples enriched in kerogen III, IV.1, and IV.2. (**A**) Content of Gram-positive and Gram-negative bacteria. (**B**) Dominating (>1%) phyla. (**C**) Dominating (>1%) classes. (**D**) Dominating (>1%) genera. The error bars represent standard deviation.

Ion Torrent™ next-generation sequencing of 16S rDNA pointed out the clear differences between the taxonomic compositions of the identified bacterial communities (Figure 5, Supplementary Information A, Figure S5 and Supplementary Information C). In the case of kerogen II samples, DNA was not isolated.

First of all, the bacterial community inhabiting kerogen III was found to be dominated by Gram-negative bacteria (82%), whereas in kerogen IV.1, the amount of Gram-negative and Gram-positive bacteria was similar (56% and 42%), and in kerogen IV.2, only Gram-positive bacteria were detected (Figure 5A).

The bacterial community inhabiting kerogen III was dominated by two phyla—*Proteobacteria* (82%) and *Firmicutes* (14%), while in kerogen IV.1, phylum *Proteobacteria* (42%) co-occurred with phylum *Actinobacteria* (56%), and in kerogen IV.2, phylum *Actinobacteria* (30%) co-occurred with phylum *Firmicutes* (69%).

The *Gammaproteobacteria* class of bacteria dominated the bacterial community in kerogen III (64%), and these organisms were represented by the genera *Acinetobacter* (32%) and *Pseudomonas* (27%). In addition, bacteria belonging to classes *Bacilli* (11%), *Alphaproteobacteria* (10%), and *Betaproteobacteria* (7%) were identified in the sample. The genera identified among these classes included *Bacillus* (9.3%), *Lactobacillus* (1.6%), *Phenylobacterium* (2.6%), and *Rhizomicrobium* (2.3%).

In kerogen IV.1, the bacterial community was dominated by *Actinobacteria* (56%) and *Gammaproteobacteria* (29%) classes. In addition, bacteria belonging to classes *Betaproteobacteria* (8.9%) and *Alphaproteobacteria* (4.4%) were observed. The identified genera of bacteria included *Pseudomonas* (7.4%), *Limnobacter* (3.2%), *Thiobacillus* (2.8%), and *Methylobacterium* (1.1%).

In the bacterial community inhabiting kerogen IV.2, two classes of bacteria were detected—*Bacilli* (69%) and *Actinobacteria* (31%), but only one genus was identified—*Bacillus* (48%).

These results highlight that although the bacterial communities identified in three types of kerogen differed in their taxonomic composition, they commonly showed the dominance of heterotrophic bacteria known for being capable of degrading aliphatic and aromatic HCs in all types of kerogen [18].

### 2.4. Bacterial Enzymes Potentially Involved in Kerogen Oxidation and Dehydrogenation

Metaproteomic studies have shown that bacterial communities inhabiting the shale rock produce enzymes involved in the aerobic degradation of organic compounds, including primary aliphatic and aromatic HCs (Supplementary Information B). In total, 51 sequences matched with 21 different enzymes capable of transforming the HCs were identified in the studied metaproteomes (Table 1). It is important to emphasize that the identified enzymes produced by bacteria were isolated, as was the DNA, directly from the rock material colonized by microorganisms, which significantly affects the number of detected sequences but is crucial to understanding processes in the mine environment.

Among the protein sequences identified, the most important were those matched with di- and monooxygenases, which oxidize organic matter. One sequence matched with catechol 1,2-dioxygenase and three sequences matched with 2-nitropropane dioxygenase (participating in the nitroalkane degradation pathway) [19] were identified in the most oxidized kerogen IV.1.

Among the monooxygenases, one sequence matched with methane monooxygenase protein B and one sequence matched with a particulate methane monooxygenase responsible for catalyzing the oxygenation of short-chain (C2–C4) HCs, which were identified in kerogen III and IV.1, respectively. In kerogen IV.1, one sequence of FAD-binding monooxygenase that catalyzes the oxygenation of long-chain (C20–C36) HCs was detected [20], while in kerogen III, one sequence of quinol monooxygenase that may oxidize benzene-1,4-diol and isoquinoline oxidoreductase involved in isoquinoline degradation [21] were identified. Moreover, one sequence of carboxymuconolactone decarboxylase involved in the metabolism of aromatic HCs in the ortho-cleavage pathway of protocatechuate [22,23] was detected in kerogen III. One sequence of another enzyme, dienelactone hydrolase, which is involved in the oxidative degradation of aromatic HCs, was identified in kerogen IV.1. It participates in the ortho-cleavage pathway of chlorocatechol (chlorobenzene-1,2-diol) formed during the degradation of chlorinated aromatic HCs [24,25].

Moreover, in kerogen III and IV.1, three sequences matching haloacid dehalogenase, an enzyme participating in the degradation pathways of chlorinated aliphatic HCs, were detected [26]. Among other important protein sequences detected in kerogen III were one sequence matching phenylacetic acid degradation protein (PaaA) and one sequence matching polyphenol oxidase (laccase). The former hydroxylates the aromatic ring of phenylacetic acid, which is an intermediate formed during the microbial degradation of aromatic compounds [27,28,29]. The latter phenol oxidase enzyme, laccase, is a copper-containing enzyme and can oxidize the polycyclic aromatic HCs in the presence of mediators, such as phenol, aniline, 4-hydroxybenzoic acid, 4-hydroxybenzyl alcohol, methionine, cysteine, and reduced glutathione [30,31].

The next group of sequences identified in the metaproteomes of bacterial communities were those matching alcohol dehydrogenases responsible for the oxidation of alcohols to aldehydes. A total of 19 sequences matched with six dehydrogenases were detected, including butanol, butanediol, cyclohexanol, and methanol dehydrogenases. These sequences were present in all the analyzed kerogen samples, but were found to be predominant in kerogen IV.1 (12 sequences matched with three alcohol dehydrogenases).

The last group of protein sequences identified in the studied metaproteomes matched with aldehyde dehydrogenases, responsible for the oxidation of aldehydes to carboxylic acids. In total, 17 sequences matched with three aldehyde dehydrogenases were detected in all the kerogen samples examined.

In addition to the above, the sequences of enzymes responsible for the degradation of fatty acids resulting from HC catabolism, including those involved in the synthesis of acetyl coenzyme A and beta-oxidation, were identified in the metaproteomes investigated (Table 1).

In summary, the presence of the detected protein sequences indicates a potential role of shale rock bacteria in kerogen oxidation and dehydrogenation. It is very important that the obtained results are consistent with the previously identified enzymes of the microbial community inhabiting weathered black shale [16], as well as with the consortium tested in laboratory conditions [15].

## 3. Materials and Methods

### 3.1. Description of the Sampling Site and Samples

This study was conducted using the shale rock of the Fore-Sudetic Monocline (Suplementary Information A, Figure S1). For testing, 6 test stands were selected from different locations of Legnicko–Głogowski Copper Mining District. The first series of samples (stand 1–2) were collected from profiles created a few days before sampling, while the rest (stands 3–6) came from old profiles that were estimated to be over 10 years old. In each stand, three samples of shale rock were taken. Samples were taken from a depth of 600–780 m below sea level. The temperature and pressure at the sampling site were between 22–26 °C and 1044–1080 hPa, respectively (Supplementary Information A, Table S1). The samples were collected aseptically from outcrops (at a depth of 20 cm) and kept at −80 °C or −4 °C until processing in the laboratory.

### 3.2. Pyrolytic Analysis

The collected samples were subjected to pyrolytic analysis using the Rock-Eval technique [6]. This technique involved the thermal decomposition of rock samples (100 mg) in two steps—pyrolysis and oxidization. In the pyrolysis step, the samples were heated at 300 °C in a nitrogen atmosphere to release the S1 fraction (mg HCs/g rock) containing volatile hydrocarbons. Following this step, the samples were heated at 650 °C to release the S2 fraction (mg HCs/g rock) measured using a flame ionization detector. The S2 fraction represented the hydrocarbons obtained due to kerogen cracking. The sum of S1 and S2 constitutes hydrocarbon potential. In the oxidation step, the samples were heated at 850 °C in an oxygen atmosphere for the release of carbon monoxide and carbon dioxide from the residual/unproductive organic and mineral matter. The results obtained from the analyses were converted into content of productive/pyrolytic organic carbon (PC), content of unproductive/residual carbon (RC), and content of TOC. The other important parameters tested using the pyrolytic analysis were HCP, HI, and OI, which allowed for characterizing the type of organic matter and the petroleum-forming potential of the examined shale rock. All analyses were performed in triplicate. The results were statistically analyzed using Student’s *t*-test. The Rock-Eval analysis of the samples was performed at the Polish Geological Institute, National Research Institute.

### 3.3. Extraction of Organic Compounds

Organic compounds were extracted from 20 g samples by a mixture of dichloromethane–methanol (vol. ratio 9:1) using automatic Soxhlet apparatus SER 158 (Velp, Italy) for 4 h (2 h of sample boiling in solvent, 30 min of solvent evaporation, 1.5 h of sample washing with a clean solvent). After extraction, the solvent was evaporated by applying an N_2_ stream, and the resulting extract was weighed. Finally, the extract was dissolved in 2 mL of dichloromethane and placed in vials of gas chromatography (GC) system equipped with teflon-coated screw caps. Each of the samples was extracted in triplicate. A blank sample was prepared according to the same procedure.

### 3.4. Analysis of Extractable Fossil Organic Matter (FOM)

The separation of organic compounds was performed using an Agilent 7890A Series Gas Chromatograph (GC) interfaced to an Agilent 5973c Network Mass Selective Detector and an Agilent 7683 Series Injector (Agilent Technologies, Santa Clara, CA, USA). A 5 µL sample was injected with split 1:5 (sample; carrier gas) by 0.3% SD to a HP-5MS column (30 m × 0.25 mm I.D., 0.25 µm film thickness, Agilent Technologies, Santa Clara, CA, USA) using He as the carrier gas at 1 mL min^−1^. The ion source was maintained at 250 °C; the GC oven was programmed with a temperature gradient starting at 100 °C (for 3 min), and this was gradually increased to 300 °C (for 5 min) at 8 °C min^−1^. Mass spectrometry analysis was carried out in the electron impact mode at an ionizing potential of 70 eV. Mass spectra were recorded from *m*/*z* 40 to 800 (0–30 min).

### 3.5. Selection, Identification, and Classification of Organic Compounds

Peaks that indicated area not less than 0.1% of the total area of total ion current chromatogram were selected for identification. The identification was performed with an Agilent Technologies Enhanced ChemStation (G1701EA ver. E.02.00.493) and The Wiley Registry of Mass Spectral Data (version 3.2, copyright 1988–2000 by Palisade Corporation with 8th Edition with Structures, copyright 2000 by John Wiley and Sons, Inc., Hoboken, NJ, USA) using a 3% cutoff threshold.

The selected peaks representing organic compounds whose mass spectra indicated compliance with reference mass spectra equal to or higher than 80% were identified. The rest of the organic compounds representing lower compliance (<80%) were assigned only to the major classes of organic compounds based on the presence of characteristic and dominating fragmentation ions (aromatic hydrocarbons—*m*/*z* 65, 77, 78, 79; aliphatic hydrocarbons—*m*/*z* 43, 57, 71, 85, 99; alcohols—*m*/*z* 45, 59, 73, 87; aldehydes—*m*/*z* 44, 58, 72; carboxylic acids—*m*/*z* 43, 45, 57, 59, 60, 71, 73, 85, 87) [32]. Those organic compounds, which were present in extracts of two repetitions of each sample, were selected for further analysis. Statistical analysis of the results was performed using Student’s *t*-test.

### 3.6. Microscopic Observations

The samples (10 mg) were fixed in paraformaldehyde vapor (21 days) and coated with gold. Then, they were observed under a scanning electron microscope SEM, Leo 1430VP (LEO Electron Microscopy Inc., Thornwood, NY, USA) at the Laboratory of Electron Microscopy (Faculty of Biology, University of Warsaw, Warsaw, Poland).

### 3.7. Carbon Metabolism Analyses

The Biolog EcoPlate™ (Biolog Inc., Hayward, CA, USA) test was used for analyzing the bacterial metabolism of the 31 most useful carbon sources (Supplementary Information D). The analyses were performed according to the manufacturer’s instructions in triplicate under aerobic, microaerobic, and anaerobic conditions. Richness index and AWCD index were calculated as described earlier by [33,34], respectively.

### 3.8. DNA Isolation

DNA was isolated according to the modified procedure of Zhou et al. [35]. A sample of 100 g was resuspended in 100 mL of DNA extraction buffer (0.1 M Na_2_EDTA, 0.1 M Tris-HCl, 0.1 M Na_2_HPO_4,_ 1.5 M NaCl, 1% hexadecyltrimethylammonium bromide (CTAB), pH 8.0) containing proteinase K and lysozyme (750 µL; 10 mg/mL) and were incubated overnight at 37 °C with horizontal shaking. Following the addition of 20% SDS, samples were incubated for 4 h at 65 °C and centrifuged (6000× *g*, 10 min). Harvested supernatants were mixed with an equal volume of chloroform–isoamyl alcohol mixture (24:1) and centrifuged (6000× *g*, 10 min). The aqueous phases were collected and precipitated overnight with 0.6 volume of isopropanol at room temperature. Next, the samples were centrifuged (16,000× *g*, 20 min, 4 °C), and the pellets were washed with 70% cold ethanol and allowed to dry. The DNA isolated and resuspended in 50 µL of sterile deionized water was stored at −80 °C.

### 3.9. DNA Amplification, Sequencing, and Bioinformatics Analysis

DNA was amplified and ligated with adapters using Ion 16S™ Metagenomics Kit according to manufacturer’s manual (Supplementary Information E). Concentration of libraries was measured on Agilent 2100 Bioanalyzer. Template for sequencing was prepared on Ion One Touch™ 2 cycler using Ion PGM™ Template OT2 400 Kit. Sequencing was performed on Ion PGM™ sequencer using Ion PGM™ Sequencing 400 Kit. The readings were analyzed with Ion Reporter™ software using metagenomics pipeline reporting all identified species (for alpha diversity and coverage analyzes) or species covered by ten reads or more (for taxonomical analyzes including the calculation of reported species). Alpha diversity factors were calculated with Ion Reporter™ software and QIIME package [36].

### 3.10. Isolation of Proteins

Proteins were isolated according to the modified procedure of Ram et al. [37]. A sample of 100 g was resuspended in 120 mL of 20 mM Tris-HCl, pH 8, shaken for 3 min, and sonicated on ice—10 × 1 min, with 1 min pauses (Sonics Vibracell; LABO PLUS, Model CV18 head). One hundred milliliters of 0.4 M Na_2_CO_3_ (pH 11) was added to the suspensions of the lysed cells, and the samples were centrifuged to remove the unlysed cells and cell membrane fragments (6000× *g*, 20 min, 4 °C), and filtered (filter with 0.22 µm pore size). Proteins were precipitated from the solution with trichloroacetic acid (1:10 (*v*/*v*)). The precipitation was performed overnight at 4 °C, and then the samples were centrifuged (20,000× *g*, 10 min, 4 °C). The aqueous phases were discarded, and the protein pellets were resuspended in 0.5 mL of methanol precooled to 4 °C, and then centrifuged (20,000× *g*, 10 min, 4 °C). The proteins were stored at −80 °C. All analyses were performed in triplicate.

### 3.11. Identification of Proteins

The identification of proteins was performed by LC-MS-MS/MS (liquid chromatography coupled to tandem mass spectrometry) using Nano-Acquity (Waters) LC system and Orbitrap Velos mass spectrometer (Thermo Electron Corp., San Jose, CA, USA) in the Institute of Biochemistry and Biophysics (Polish Academy of Science, Warsaw, Poland). The equipment used was sponsored in part by the Centre for Preclinical Research and Technology (CePT), a project co-sponsored by European Regional Development Fund and Innovative Economy, The National Cohesion Strategy of Poland.

Prior to the analysis, proteins were subjected to standard “in-solution digestion” procedure, during which proteins were reduced with 50 mM Tris (2-carboxyethyl)phosphine (for 60 min at 60 °C), alkylated with 200 mM S-methyl methanethiosulfonate (45 min at room temperature), and digested overnight with trypsin (Sequencing Grade Modified Trypsin; Promega V5111). Peptide mixture was injected on RP-18 precolumn (nanoACQUITY Symmetry^®^ C18; Waters 186003514) using water containing 0.1% trifluoroacetic acid as mobile phase and then transferred to nano-HPLC RP-18 column (nanoACQUITY BEH C18; Waters 186003545) using an acetonitrile (ACN, 5–35% in 180 min) in the presence of 0.05% formic acid at the flow rate of 250 µL/min. Column outlet was directly coupled to the ion source of the spectrometer working in the regime of data-dependent MS to MS/MS switch. A blank run ensuring the lack of cross contamination from previous samples preceded each analysis.

Acquired raw data were processed by Mascot Distiller software, followed by a Mascot search (Matrix Science, London, UK, on-site license) in the NCBInr database. Peptides with Mascot score exceeding the threshold value corresponding to <5% expectation value, calculated by Mascot, were considered to be positively identified. Metaproteomes analysis was performed using GhostKOALA and automatic annotation and KEGG mapping service according to Kanehisa et al. [38].

## 4. Conclusions

The results of the work presented confirm previous reports [16,17] and, at the same time, significantly broaden our knowledge on postdiagenetic kerogen biotransformation. Based on the results of current field studies, but also in connection with previous experimental studies [16], we can conclude that the type and HCP of kerogen are influenced not only by its precursors and the conditions of sedimentation/diagenesis occurring in previous geological periods but also by the bacterial communities currently inhabiting the shale rocks. The obtained results indicate that the primary type of kerogen, which is characteristic for the deposit, can evolve in different ways (Kerogen III, IV.1, and IV.2). In addition, we have shown that the oxidation and dehydrogenation processes potentially carried out by bacteria can deprive the kerogen of its ability to generate both liquid and gaseous HCs. At the same time, these changes do not have to be associated with strong kerogen oxidation, only with dehydrogenation. Furthermore, we have revealed that the weathering processes involving various bacterial communities, as well as those occurring in different conditions, can contribute to a significant diversity of kerogen on the earth.

Finally, it is worth adding that the processes described in this paper show the activation of fossil organic carbon buried for millions of years in the sedimentary rocks and its probable incorporation into the global cycle on the earth. Thus, taking into account the number of shale rocks present on earth that contain buried fossil organic matter, it can be concluded that the described processes may be of global significance.

## Figures and Tables

**Table 1:** Sequences of bacterial enzymes potentially involved in the oxidation and dehydrogenation of kerogen as well as in the metabolism of fatty acids, detected in the three studied bacterial communities (kerogen III, IV.1, and IV.2).

Enzyme	Kerogen III	Kerogen IV.1	Kerogen IV.2
Dioxygenases			
Catechol 1,2-dioxygenase	-	gi|498129511	-
2-Nitropropane dioxygenase	-	gi|490244293 gi|496990711 gi|490758414	-
Monooxygenases			
Methane monooxygenase protein B	gi|223717934	-	-
Particulate methane monooxygenase B-subunit	-	gi|306921967	-
FAD-binding monooxygenase	-	gi|517722695	-
Quinol monooxygenase	gi|501096931	-	-
Other oxidizing enzymes			
Carboxymuconolactone decarboxylase	gi|488115986	-	-
Dienelactone hydrolase	-	gi|490775052	-
Haloacid dehalogenase	gi|503054342 gi|503823017	gi|544647509	-
Phenylacetic acid degradation protein	gi|523636522	-	-
Isoquinoline 1-oxidoreductase subunit alpha	gi|522028532	-	-
YfiH family protein (laccase)/polyphenol oxidase	gi|523636522	-	-
Alcohol dehydrogenases			
Alcohol dehydrogenase	-	gi|446065336 gi|481050357 gi|490834704 gi|491126456 gi|517727322 gi|489375334 gi|489392338 gi|490768969 gi|498009882 gi|499606054	WP_004631200.1 WP_011286788.1
Alkyl alcohol (butanol) dehydrogenase	gi|283480587	-	gi|283480587
(R,R)-butanediol dehydrogenase	-	gi|494048403	-
Cyclohexanol dehydrogenase	-	gi|9965291	-
Lanthanide-dependent methanol dehydrogenase	-	-	gi|177826798, gi|495339880
Methanol dehydrogenase	gi|492375028	-	-
Aldehyde dehydrogenases			
Acetaldehyde dehydrogenase	-	gi|494200545	-
Aldehyde dehydrogenase	gi|430004500 gi|742727449 gi|501570423 gi|499520282	gi|518406973 gi|489377479 gi|490789497 gi|491118422 gi|491126465 gi|493682336 gi|500262322 gi|518406973 gi|635596374	WP_0195771801 BAO81531.1
Phenylacetaldehyde dehydrogenase	CAA67780.1		
Enzymes participating in degradation of fatty acids			
Acyl-CoA synthesis			
Long-chain-fatty-acid-CoA ligase	WP_003869413.1 WP_007704349.1	gi|371548208| gb|EHN76535.1| gi|493470804| |	-
Beta-oxidation			
Acyl-CoA oxidase	sp|Q9Y7B1.1	-	-
Enoyl-CoA hydratase	WP_016918268.1 WP_029919295.1	-	WP_016918268.1
3-hydroxyacyl-CoA dehydrogenase	WP_014612827.1	gi|504425725	-
Medium-chain acyl-CoA dehydrogenase	-	gi|501277187	-
Other enzymes			
Acetyl-CoA C-acetyltransferase	AAG30258.1	-	WP_011531005.1
Glutaryl-CoA dehydrogenase (ETF)	-	-	WP_018763741.1

## Data Availability

Not applicable.

## Supplementary Materials

The following are available online at https://www.mdpi.com/article/10.3390/molecules27082408/s1. Supplementary Information A. Supplementary figures and table. Supplementary Information B. Metaproteomes of bacteria inhabiting shale rock. Supplementary Information C. Taxonomic diversity of bacteria inhabiting shale rock. Supplementary Information D. The Biolog EcoPlate™Supplementary Information E. Ion 16S™ Metagenomics Kit.

## Sample Availability

Not available.
