# Supplementary material for: Postdiagenetic Changes in Kerogen Properties and Type by Bacterial Oxidation and Dehydrogenation
Source: Molecules. 2022 Apr 8;27(8):2408. doi: 10.3390/molecules27082408 (PMC9030554; doi:10.3390/molecules27082408)
Supplement: Supplementary file 1 [file molecules-27-02408-s001.zip › Supplementary Information A_Supplementary Figures and Table.pdf]

Supplementary Information A.

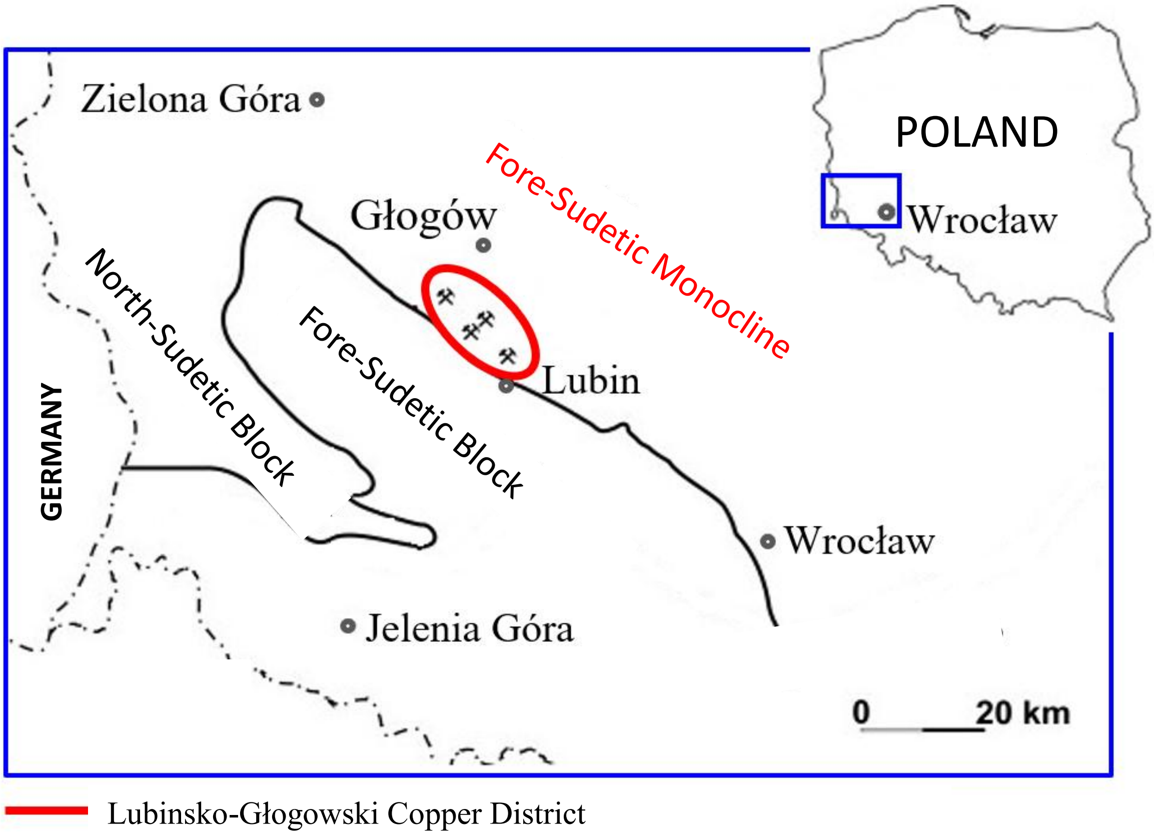

Figure S1. Localization of Fore-Sudetic Monocline and Lubinsko-Głogowski Copper District (red circle)

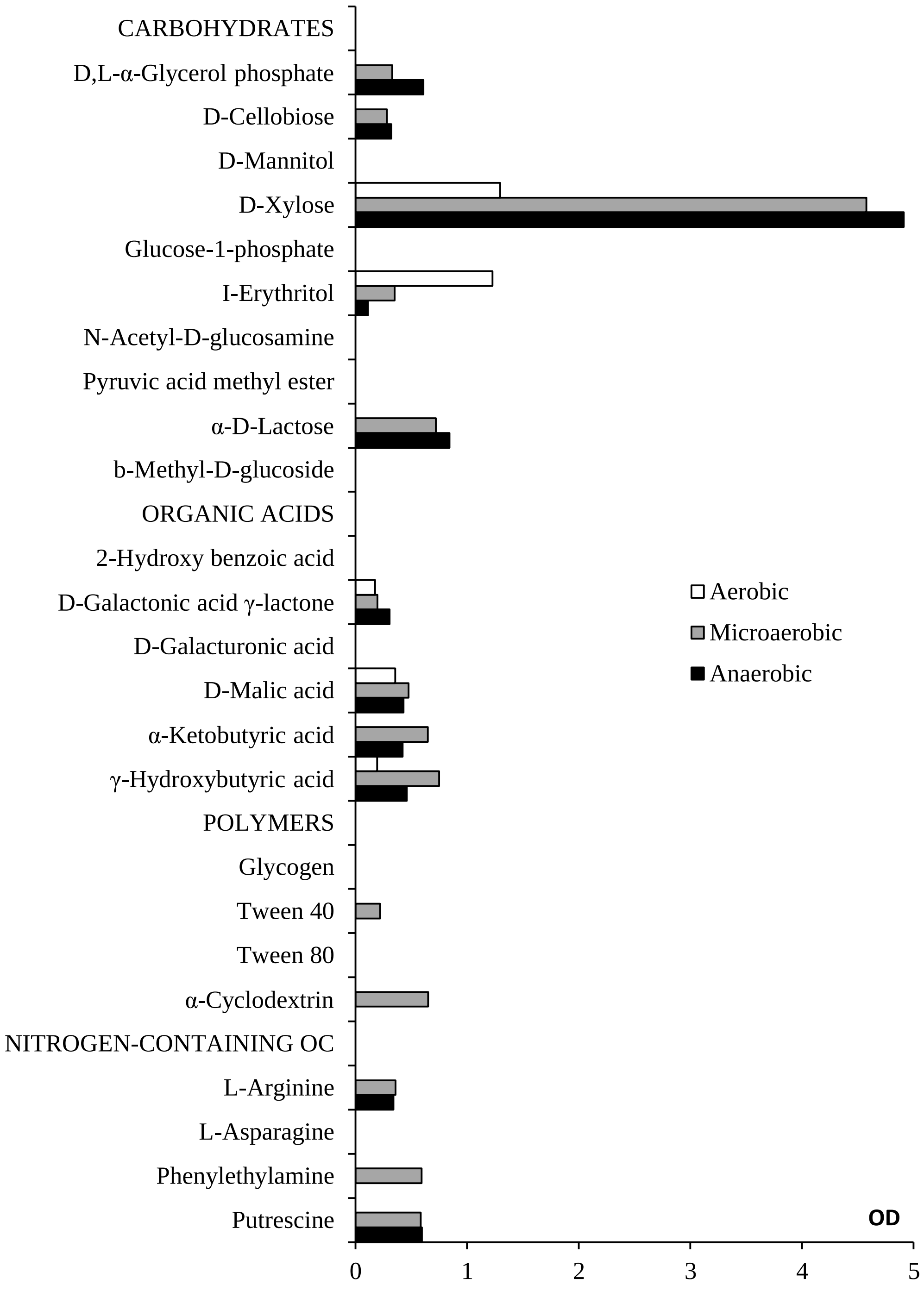

Figure S2. Metabolic activity of microorganisms inhabiting shale rock enriched in kerogen III under aerobic, microaerophilic and anaerobic conditions; the optical density (OD) values correspond to the degradation intensity of organic compounds; OC – organic compounds

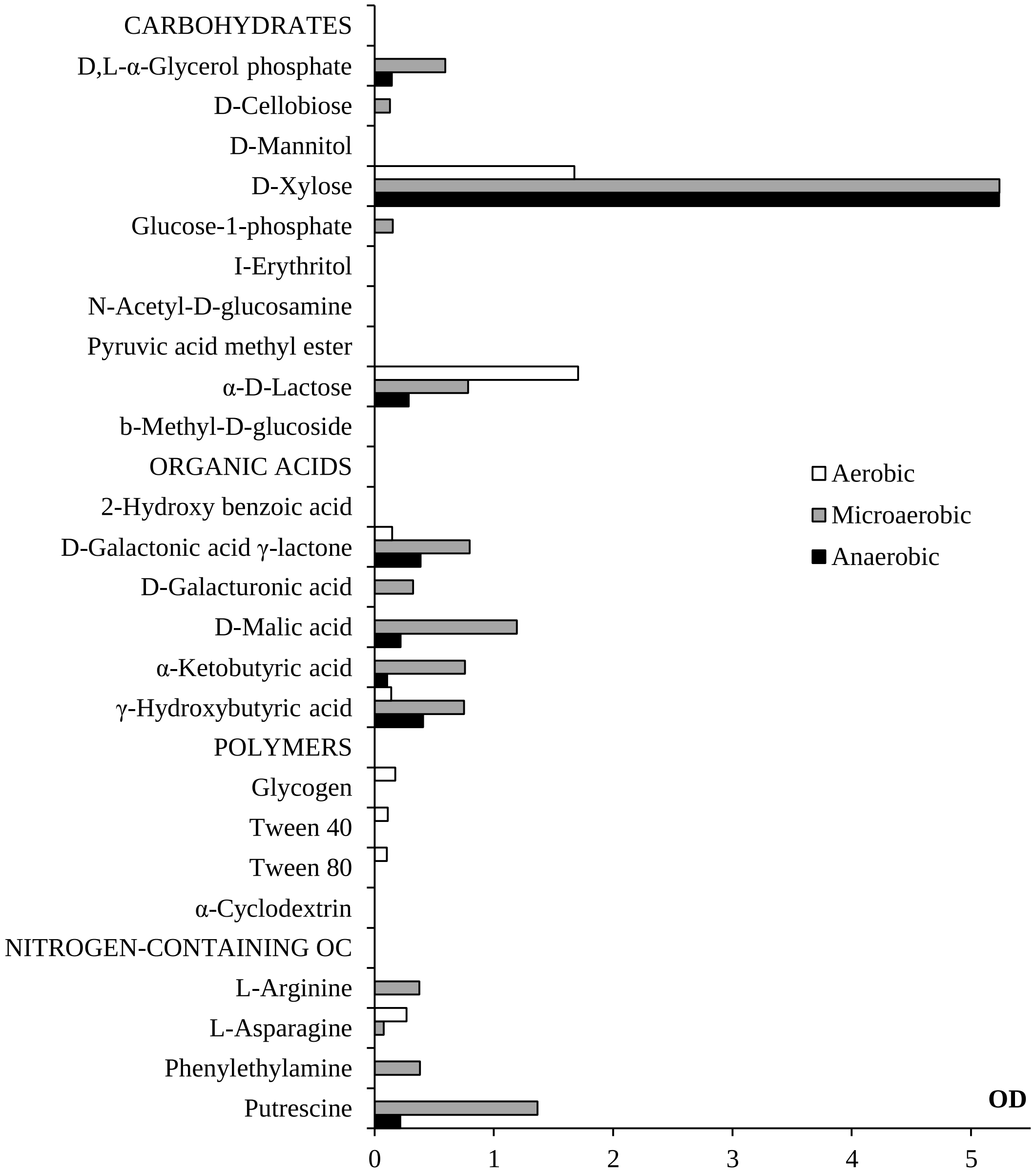

Figure S3. Metabolic activity of microorganisms inhabiting shale rock enriched in kerogen IV.1 under aerobic, microaerophilic and anaerobic conditions; the optical density (OD) values correspond to the degradation intensity of organic compounds; OC – organic compounds

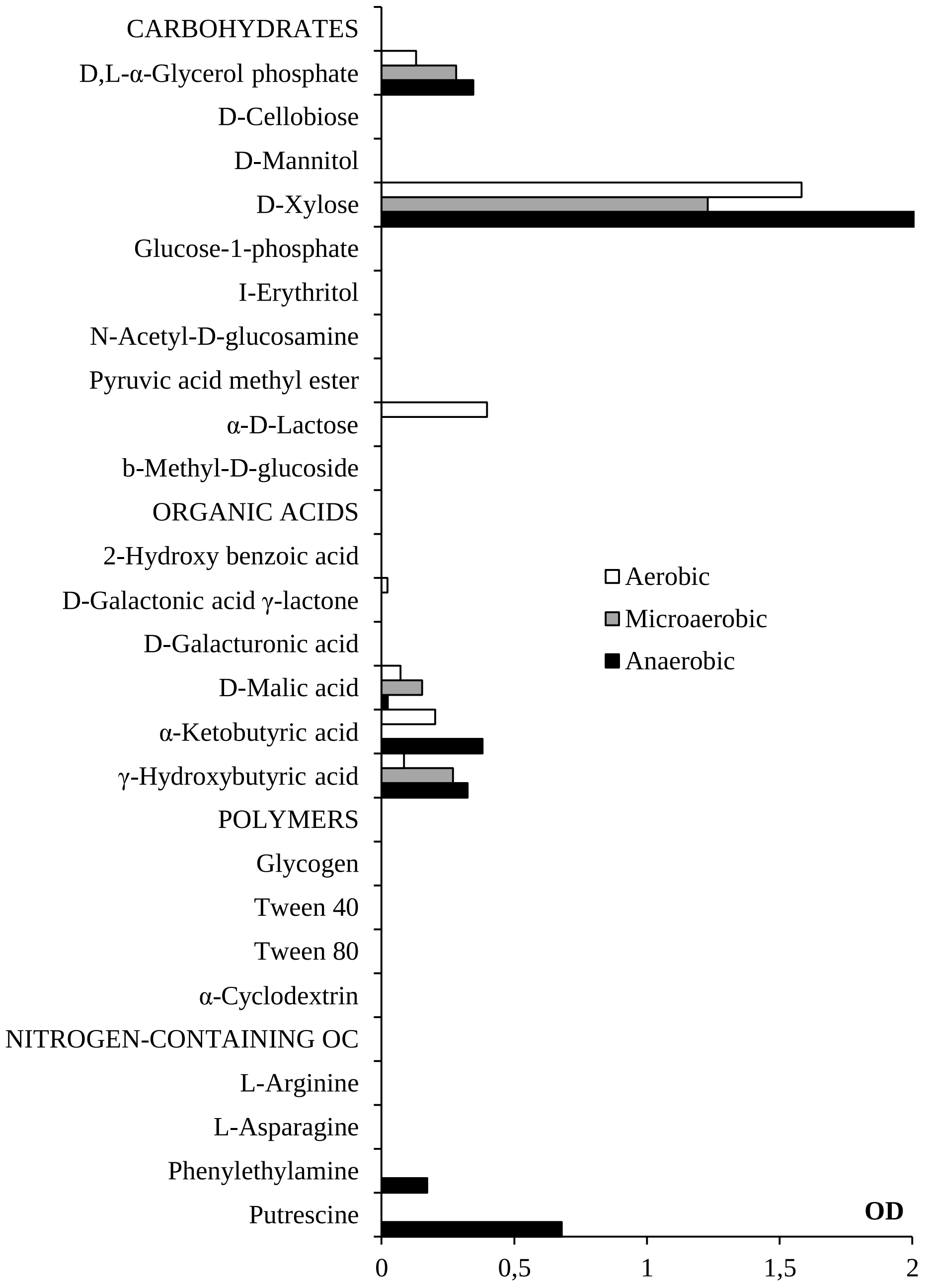

Figure S4. Metabolic activity of microorganisms inhabiting shale rock enriched in kerogen IV.2 under aerobic, microaerophilic and anaerobic conditions; the optical density (OD) values correspond to the degradation intensity of organic compounds; OC – organic compounds

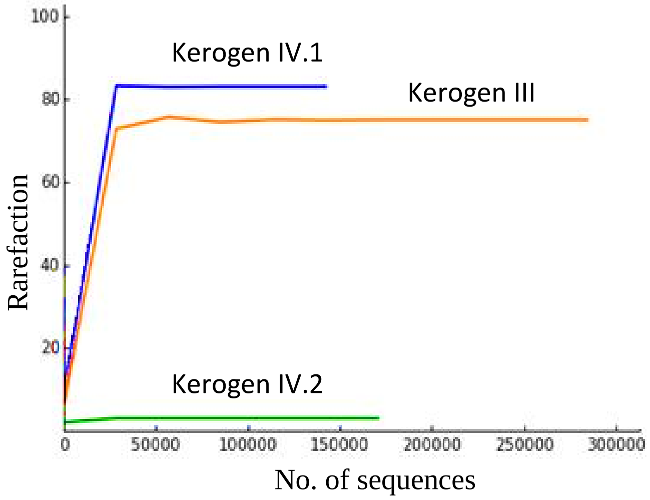

Figure S5. Rarefaction curve of 16S rDNA sequencing of three samples (kerogen III, IV.1 and IV.2)

Table S1. Characteristics of research stands where shale rock samples were collected

**Parametr**	**Research stand**				
	**Stand 1**	**Stand 1**	**Stand 3**	**Stand 4**	**Stand 5**
Depth (m)	600	650	700	780	650
Temperature	22	23	26	22	25
Pressure (hPa)	1044	1065	1080	1070	1067
