# Supplementary material for: Postdiagenetic Changes in Kerogen Properties and Type by Bacterial Oxidation and Dehydrogenation
Source: Molecules. 2022 Apr 8;27(8):2408. doi: 10.3390/molecules27082408 (PMC9030554; doi:10.3390/molecules27082408)
Supplement: Supplementary file 1 [file molecules-27-02408-s001.zip › Supplementary Information D_ Biolog EcoPlate Methods.pdf]

EcoPlate™

A rapid, community-level assessment tool for studying microbial community dynamics

Biolog EcoPlates provide a sensitive and reliable index of environmental change. This approach, called community-level physiological profiling (CLPP), is effective at distinguishing spatial and temporal changes in the metabolism of microbial communities.

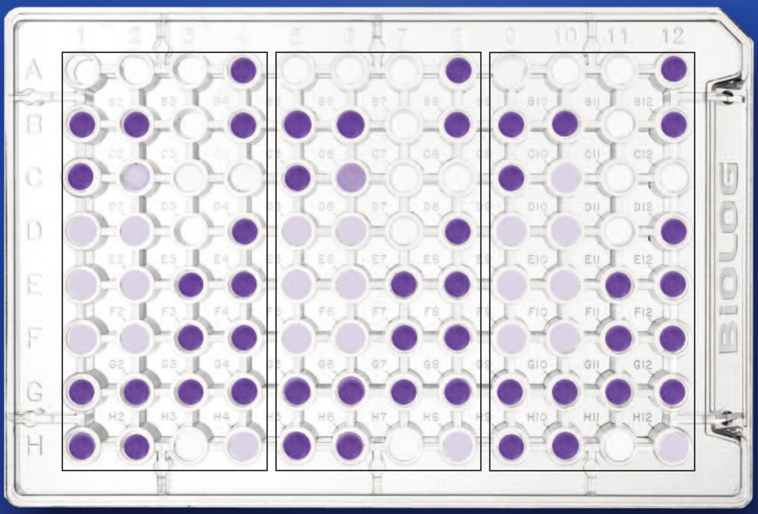

31 carbon assays in triplicate

EcoPlates contain 3 repeated sets of 31 carbon sources and employ a tetrazolium redox dye as an indicator of microbial metabolism. As microbes utilize the carbon sources they respire and the tetrazolium reporter dye is reduced to form a visible purple color. Communities of microorganisms will exhibit a characteristic reaction pattern, a metabolic fingerprint, that reflects the metabolic properties of the community.

EcoPlates can be used in many applications including:

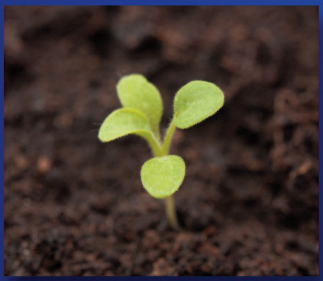

Analyzing population changes in soil

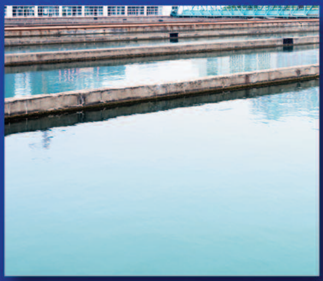

Water and wastewater profiling

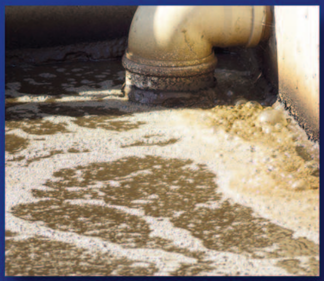

Activated sludge, compost & industrial waste testing

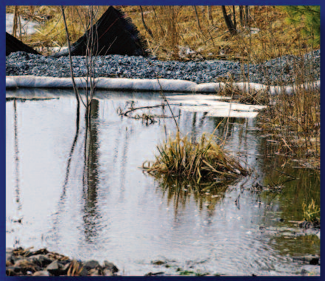

Monitoring bioremediation and effects of toxic chemicals

BIOLOG, Inc.
21124 Cabot Blvd
Hayward, CA 94545 USA
(510) 785-2564 · www.biolog.com

BIOLOG

31 Carbon Source Measured Assays:

β-Methyl-D-Glucoside	D-Galactonic Acid γ-Lactone	L-Arginine
Pyruvic Acid Methyl Ester	D-Xylose	D-Galacturonic Acid
L-Asparagine	Tween 40	i-Erythritol
2-Hydroxy-Benzoic Acid	L-Phenylalanine	Tween 80
D-Mannitol	4-Hydroxy-Benzoic Acid	L-Serine
α-Cyclodextrin	N-Acetyl-D-Glucosamine	γ-Hydroxy-Butyric Acid
L-Threonine	Glycogen	D-Glucosaminic Acid
Itaconic Acid	Glycyl-L-Glutamic Acid	D-Cellobiose
Glucose-1-Phosphate	α-Keto-Butyric Acid	Phenylethylamine
α-D-Lactose	D,L-α-Glycerol-Phosphate	D-Malic Acid
Putrescine		

Key Applications:

- Community-level physiological profiling (CLPP)
- Monitoring temporal or spatial changes in microbial community activity
- Observing changes before and after perturbation
- Metabolic community fingerprinting
- Screening community diversity / similarity
- Assaying Carbon Source Utilization Patterns (CSUP)
- Monitoring changes in community functional diversity

In applied ecological research, EcoPlates are used as both an assay of the stability of a normal population and to detect and assess changes following the onset of an environmental variable.

The utility of using Biolog technology to analyze microbial communities has been documented in over 500 publications. Visit our bibliography at www.biolog.com

Ordering Information:

Catalog #1506 - EcoPlate (box of 10)

Typical Procedure:

STEP 1: Environmental samples (as aqueous samples or after suspension) are diluted to a standard cell density and then pipetted directly into EcoPlates.

STEP 2: EcoPlates are incubated and the metabolic pattern formation of the community is recorded kinetically.

STEP 3: The community-level physiological profile is assessed for key characteristics such as pattern stability (similarity), rate of color change in each well (activity), and richness of well response (diversity).

EcoPlate Data Collection:

EcoPlates should be read kinetically to capture the profile of substrate metabolism. This can be accomplished with repeated reading of microplates using a microplate reader such as the MicroStation™. A better option, especially when many samples are being analyzed, is the fully automated OmniLog® incubator/reader which automatically reads up to 50 EcoPlates every 15 minutes. The MicroStation and OmniLog come with software that records all data and facilitates analysis.

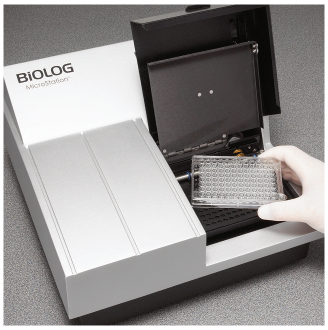

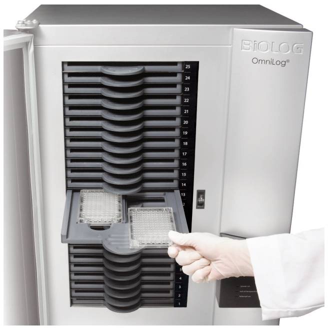

Part 00A 068, Rev A 11/2017

BIOLOG, Inc.
21124 Cabot Blvd
Hayward, CA 94545 USA
(510) 785-2564 · www.biolog.com

BIOLOG
