# Supplementary material for: Postdiagenetic Changes in Kerogen Properties and Type by Bacterial Oxidation and Dehydrogenation
Source: Molecules. 2022 Apr 8;27(8):2408. doi: 10.3390/molecules27082408 (PMC9030554; doi:10.3390/molecules27082408)
Supplement: Supplementary file 1 [file molecules-27-02408-s001.zip › Supplementary Information E_Ion_16S_Methods.pdf]

USER GUIDE

ion torrent

Ion 16S™ Metagenomics Kit

Catalog Number A26216

Publication Number MAN0010799

Revision C.0

For Research Use Only. Not for use in diagnostic procedures.

Thermo Fisher SCIENTIFIC

The information in this guide is subject to change without notice.

DISCLAIMER

TO THE EXTENT ALLOWED BY LAW, LIFE TECHNOLOGIES AND/OR ITS AFFILIATE(S) WILL NOT BE LIABLE FOR SPECIAL, INCIDENTAL, INDIRECT, PUNITIVE, MULTIPLE, OR CONSEQUENTIAL DAMAGES IN CONNECTION WITH OR ARISING FROM THIS DOCUMENT, INCLUDING YOUR USE OF IT.

Important Licensing Information

These products may be covered by one or more Limited Use Label Licenses. By use of these products, you accept the terms and conditions of all applicable Limited Use Label Licenses.

Trademarks

All trademarks are the property of Thermo Fisher Scientific and its subsidiaries unless otherwise specified. AmpErase is a trademark of Roche Molecular Systems, Inc. TaqMan is a registered trademark of Roche Molecular Systems, Inc., used under permission and license. Bioanalyzer and Agilent are trademarks of Agilent Technologies, Inc. Agencourt and AMPure are trademarks of Beckman Coulter, Inc. Eppendorf LoBind and Eppendorf are trademarks of Eppendorf AG. Adobe and Reader are trademarks of Adobe Systems Incorporated.

©2015 Thermo Fisher Scientific Inc. All rights reserved.

Contents

About this guide 5

Revision history 5

Product information 6

Product description 6
Kit contents 6
Required materials and equipment (not provided) 7

Methods 9

Workflow 9
Prepare amplicons 10
Important sample preparation and amplification guidelines 10
Amplify the 16S hypervariable regions 11
Prepare reagents for purification 11
Purify the amplification products in the PCR plate 12
Purify the amplification products in tubes 13
Calculate DNA input for library preparation 14
Prepare the library 14
End repair and purify pooled amplicons 14
Ligate and nick-repair 16
Purify the adapter-ligated and nick-repaired DNA 17
Determine library concentration 18
Determine library concentration using qPCR 19
Determine library concentration using a Bioanalyzer® instrument 22
Proceed to template preparation 25
Sequence the library 25

■ **APPENDIX A** Troubleshooting 26

■ **APPENDIX B** Example: Calculate DNA input for library preparation 27

■ **APPENDIX C** Good laboratory practices for PCR and RT-PCR 28

■ **APPENDIX D** Safety 29

Chemical safety 30
Biological hazard safety 31

Documentation and support 32

Related documentation 32
Customer and technical support 32
Limited product warranty 33

About this guide

IMPORTANT! Before using this product, read and understand the information in the "Safety" appendix in this document.

Revision history

Revision	Date	Description
C.0	June 2015	Updated the template preparation and sequencing kits supported for use with the Ion 16S™ Metagenomics Kit:<br>• Added the Ion PGM™ Hi-Q™ OT2 Kit and Ion PGM™ Hi-Q™ Sequencing Kit.<br>• Added template preparation instructions for use with the Ion PGM™ Hi-Q™ OT2 Kit (see "Proceed to template preparation" on page 25).<br>• Removed references to the Ion PGM™ Template OT2 400 Kit and Ion PGM™ Sequencing 400 Kit.
B.0	October 2014	Updated the thermal cycling conditions in "Run the real-time PCR reactions" on page 21.

Product information

Product description

The Ion 16S™ Metagenomics Kit is designed for rapid analyses of polybacterial samples using Ion Torrent™ sequencing technology. The kit includes two primer sets that selectively amplify the corresponding hypervariable regions of the 16S region in bacteria:

- Primer set V2-4-8
- Primer set V3-6, 7-9

Sequence the amplified fragments on the Ion PGM™ System and analyze the results with the Ion Reporter™ software Ion 16S™ Metagenomics Kit analyses module. The combination of the two primer pools allows for sequence-based identification of a broad range of bacteria within a mixed population.

Kit contents

Table 1 Ion 16S™ Metagenomics Kit (Cat. no. A26216)

Component	Part no.[1]	Cap color	Quantity	Volume	Storage conditions
2X Environmental Master Mix	4401975	Orange	2 tubes	0.8 mL per tube	Shipped at –20°C.<br>After first use, store at 2–8°C if used frequently, otherwise store at –20°C.<br>Protect the 2X Environmental Master Mix from light.
DNA Dilution Buffer	4405587	Clear	1 bottle	7.0 mL	
16S Primer Set V2–4–8 (10X)	100026495	Green	1 tube	300 µL	Store at –15 to –25°C.
16S Primer Set V3–6, 7–9 (10X)	100026496	Blue	1 tube	300 µL	
Negative Control	362250	White	1 tube	1.0 mL	
E. coli DNA control (30 µg/mL)	4458450	Red	1 tube	40 µL	Store at –15 to –25°C.<br>Store separately from other reagents to prevent cross-contamination.

[1] Provided for identification purposes; the parts cannot be ordered separately from the kit.

Required materials and equipment (not provided)

Table 2 Ion Universal Library Quantitation Kit (Cat. no. A26217)

Component	Part no.[1]	Cap color	Quantity	Volume	Storage conditions
TaqMan® Fast Universal PCR Master Mix (2X), no AmpErase® UNG	4352046	Red	2 tubes	1.43 mL per tube	Shipped at –20°C.<br>After first use, store at 2–8°C if used frequently, otherwise store at –20°C.
Ion Library TaqMan® Quantitation Assay, 20X	4468529	White	1 tube	250 µL	–20°C
E. coli DH10B Ion Control Library	4468526	Yellow	2 tubes	25 µL per tube	–20°C

[1] Provided for identification purposes; the parts cannot be ordered separately from the kit.

Note: The Ion Universal Library Quantitation Kit is required if you use qPCR to determine the library concentration.

Table 3 Additional Ion kits used with the Ion 16S™ Metagenomics Kit

Description	Cat. no.[1]	Quantity
Ion Plus Fragment Library Kit	4471252	1 kit
Ion Xpress™ Barcode Adapters 1-16 Kit[2]	4471250	1 kit
Ion PGM™ Hi-Q™ OT2 Kit	A27739	1 kit
Ion PGM™ Enrichment Beads	4478525	1 kit
Ion PGM™ Hi-Q™ Sequencing Kit (use with Ion PGM™ Hi-Q™ Wash 2 Bottle Kit)	A25592 and A25591	1 kit

[1] All materials are available from www.lifetechnologies.com.
[2] Additional barcodes are available from www.lifetechnologies.com.

Table 4 Ion Chip kits compatible with the Ion 16S™ Metagenomics Kit

Component	Quantity	Catalog no.	Storage
Ion 318™ Chip Kit v2	4 pack<br>8 pack	4484354<br>4484355	15°C to 30°C
Ion 318™ Chip v2 BC	4 pack<br>8 pack	4488146<br>4488150	
Ion 316™ Chip Kit v2	4 pack<br>8 pack	4483188<br>4483324	
Ion 316™ Chip v2 BC	4 pack<br>8 pack	4488145<br>4488149	

Component	Quantity	Catalog no.	Storage
Ion 314™ Chip Kit v2	8 pack	4482261	15°C to 30°C
Ion 314™ Chip v2 BC	8 pack	4488144	

Table 5 Other required materials and equipment

Description	Cat. no.[1]	Quantity
Ion Personal Genome Machine™ (PGM™) System	4462921	1 system
GeneAmp™ PCR System 9700 or other thermal cycler	4314879 or MLS[2]	1 system
Agilent® 2100 Bioanalyzer® instrument (preferred)<br>*or*<br>Qubit™ 2.0 Fluorometer	Agilent Technologies G2939AA<br><br>Q32866	1 instrument
Quant-iT™ dsDNA Assay Kit, High Sensitivity (for fluorescent plate readers or Qubit™ 2.0 Fluorometer)<br>*or*<br>Qubit™ dsDNA HS Assay Kit	Q33120<br><br>Q32851	1 kit
Agencourt® AMPure® XP Kit	Beckman Coulter A63880 or A63881	1 kit
Agilent®High Sensitivity DNA Kit	Agilent Technologies 5067-4626	1 kit
1.5-mL Eppendorf® DNA LoBind® Tubes	Eppendorf 022431021	1 box
DynaMag™-2 magnet (magnetic rack; for 1.5-mL Eppendorf® tubes)	12321D	1 rack
DynaMag™-96 Bottom Magnet (for 96-well plates and 0.2-mL PCR tubes)	12332D	1 magnet
0.2-mL PCR tubes or 96-well plates	Select tubes or plates compatible with your thermal cycler (Life Technologies or MLS[2])	—
Nuclease-Free Water	AM9939 or MLS[2]	—

[1] Unless otherwise indicated, all materials are available from **www.lifetechnologies.com**.
[2] MLS: Fisher Scientific (**www.fisherscientific.com**) or other major laboratory supplier.

Methods

Workflow

The required Ion kit is listed next to the applicable step(s).

**Prepare amplicons**	
Amplify the 16S hypervariable regions	Ion 16S™ Metagenomics Kit (Cat. no. A26216)
▼	
Purify the amplification products (in a PCR plate or tubes)	
▼	
Calculate DNA input for library preparation	
▼	
**Prepare the library**	Ion Plus Fragment Library Kit (Cat. no. 4471252)
End repair and purify pooled amplicons	
▼	
Ligate and nick-repair	
▼	
Purify the adapter-ligated and nick-repaired DNA	
▼	
**Determine library concentration**	Ion Universal Library Quantitation Kit (Cat. no. A26217)
▼	
**Proceed to template preparation**	Ion PGM™Hi-Q™ OT2 Kit (Cat. no. A27739)
▼	
**Sequence the library**	Ion PGM™ Hi-Q™ Sequencing Kit (Cat. no. A25592)

Prepare amplicons

Important sample preparation and amplification guidelines

- Prepare genomic DNA using your method of choice.
- See the table for recommended DNA input amounts and number of PCR cycles; you can increase or decrease the number of PCR cycles as needed depending on your DNA input amount and sample complexity.

IMPORTANT! Minimize the number of amplification cycles to avoid over-amplification, production of concatemers, and introduction of PCR-induced errors.

- Include a positive and negative amplification control for each PCR run.
- Review Appendix C, "Good laboratory practices for PCR and RT-PCR".

Note: Throughout the user guide, "room temperature" is 22–24°C.

Table 6 PCR amplification guidelines

Sample	DNA input amount for amplification reaction[1]	No. amplification cycles
Pure microbial DNA (including positive control)	1–3 ng	18
Samples with large amounts of non-microbial DNA[2]	1–2 µL extracted DNA	25

[1] 1–3 ng of microbial DNA input provides sufficient amplified material for library preparation, and allows you to avoid reamplifying the library before the template preparation and sequencing steps.

[2] Recommended if non-microbial DNA makes it difficult to assess the microbial DNA content. See Appendix A, "Troubleshooting" for details.

Amplify the 16S hypervariable regions

See "Important sample preparation and amplification guidelines" on page 10 for guidelines on input amount and cycle number.

1. Thaw all Ion 16S™ Metagenomics Kit reagents and keep on ice.

2. For each sample, prepare two reactions (one for each of the 2 primer sets). Include one positive and negative control per PCR run. Before you pipet each reagent, vortex for 5 seconds and pulse-spin the reagent tube.

Component	Sample or positive control volume	Negative control volume
2X Environmental Master Mix	15 µL	15 µL
16S Primer Set (10X)[1]	3 µL	3 µL
DNA (sample or diluted *E. coli* DNA control)	2–12 µL sample *or* 2 µL diluted control[2]	N/A
Negative Control (water)	to 30 µL	to 30 µL
**Total**	**30 µL**	**30 µL**

[1] V2-4-8 or V3-6, 7–9
[2] Dilute the *E. coli* DNA control stock 1:20 (1.5 ng/µL) with DNA Dilution Buffer. Use 2 µL of the diluted DNA control (3 ng DNA input) in the positive control reaction.

3. Place the tubes or plate in the thermal cycler and run the following program:

Stage	Temperature	Time
Hold	95°C	10 min
Cycle 18–25 cycles[1]	95°C	30 sec
	58°C	30 sec
	72°C	20 sec
Hold	72°C	7 min
Hold	4°C	∞[2]

[1] See "Important sample preparation and amplification guidelines" on page 10.
[2] Remove samples within 24 hours and continue to next step or store at –20°C for up to 2 weeks.

4. *(Optional)* If samples contain non-microbial DNA, confirm the presence of PCR products (use a Bioanalyzer® instrument or 2% agarose gel) before you continue to the purification step. If no PCR products are present, see Appendix A, "Troubleshooting".

Prepare reagents for purification

1. Allow the Agencourt® AMPure® XP beads to come to room temperature (~30 minutes).

2. Prepare 70% ethanol. Store in a tightly closed container at room temperature when not in use.

IMPORTANT! Always use 70% ethanol for the next steps. A higher percentage of ethanol causes inefficient washing of smaller-sized molecules. A lower percentage of ethanol may cause sample loss.

Continue to “Purify the amplification products in the PCR plate“ on page 12 or “Purify the amplification products in tubes“ on page 13.

Purify the amplification products in the PCR plate

Note: If you choose to purify the amplification products in the PCR plate, you will pool the amplicons from the V2-4-8 and V3-6, 7–9 primer reactions before you perform “Calculate DNA input for library preparation“ on page 14.

1. Pulse-spin the plate. Vortex the Agencourt® AMPure® XP Reagent to resuspend, add 54 µL (or 1.8 × *sample volume*) to each 30 µL sample, then pipet up and down 5 times to thoroughly mix.

2. Incubate the mixture for 5 minutes at room temperature.

3. Place the plate in a magnetic rack such as the DynaMag™-96 Bottom Magnet for 3 minutes or until the solution is clear. Carefully remove and discard the supernatant without disturbing the bead pellet.

4. Without removing the plate from the magnet, add 100 µL of 70% ethanol, then incubate for 30 seconds. After the solution clears, remove and discard the supernatant without disturbing the pellet.

5. Repeat step 4 for a second wash.

6. To remove residual ethanol, keep the plate on the magnetic rack and carefully remove any remaining supernatant with a 20-µL pipettor without disturbing the pellet.

7. Keeping the plate on the magnetic rack, air-dry the beads at room temperature for 4 minutes.

   IMPORTANT! Do not let the pellet dry out completely.

8. Remove the plate from the magnetic rack and add 15 µL of Nuclease-free Water directly to the pellet to disperse the beads. Mix thoroughly by pipetting the suspension up and down 5 times or more as needed to resuspend the beads.

9. Place the plate in the magnetic rack for at least 1 minute until the solution clears. Transfer the supernatant containing the eluted DNA to a new PCR plate or tube without disturbing the pellet.

   IMPORTANT! The supernatant contains your sample. **Do not discard.**

STOPPING POINT *(Optional)* Store the DNA at –30°C to –10°C for up to 2 weeks.

Continue to “Calculate DNA input for library preparation“ on page 14.

Purify the amplification products in tubes

1. Combine the following in an Eppendorf® tube. Vortex the Agencourt® AMPure® XP Reagent to resuspend before taking aliquots.

Component	Volume	Example
Pooled amplification reaction[1]	X<br>(equal volumes of V2-4-8 and V3-6, 7–9 reactions)	40 µL<br>(20 µL each of V2-4-8 and V3-6, 7–9 reactions)
Agencourt® AMPure® XP Reagent	1.8X	72 µL

[1] Combine equal volumes of the 2 reactions. Alternatively, purify the 2 reactions separately, then combine equal volumes before you perform "Calculate DNA input for library preparation" on page 14.

2. Vortex the mixture briefly, pulse-spin, then incubate the mixture at room temperature for 5 minutes.

3. Pulse-spin and place the tubes in a magnetic rack such as the DynaMag™-2 magnet for 3 minutes or until the solution is clear. Carefully remove and discard the supernatant without disturbing the bead pellet.

4. Without removing the tubes from the magnetic rack, add 300 µL of 70% ethanol to each tube. Incubate for 30 seconds, turning the tubes around twice in the magnet to move the beads around. After the solution clears, remove and discard the supernatant without disturbing the pellet.

5. Repeat step 4 for a second wash.

6. To remove residual ethanol, pulse-spin the tube, place it back in the magnetic rack, and carefully remove any remaining supernatant with a 20-µL pipettor without disturbing the pellet.

7. Keeping the tube on the magnetic rack, air-dry the beads at room temperature for 4 minutes.

   IMPORTANT! Do not let the pellet dry out completely.

8. Remove the tube from the magnetic rack and add 15 µL of Nuclease-free Water directly to the pellet to disperse the beads. Vortex the sample for 5–10 seconds as needed to resuspend the beads.

9. Pulse-spin and place the tube in the magnetic rack for at least 1 minute until the solution clears. Transfer the supernatant containing the eluted DNA to a new 1.5-mL Eppendorf LoBind® Tube without disturbing the pellet.

   IMPORTANT! The supernatant contains the eluted DNA. **Do not discard.**

STOPPING POINT *(Optional)* Store the DNA at –30°C to –10°C for up to 2 weeks.

Continue to "Calculate DNA input for library preparation" on page 14.

Calculate DNA input for library preparation

1. If you did not combine equal volumes of V2-4-8 and V3-6, 7–9 amplification reactions previously, combine equal volumes of the purified DNA product for use in the following step and in library preparation.

2. Analyze the purified PCR product using one of the following methods:

Method	Procedure
Agilent® 2100 Bioanalyzer® instrument with Agilent® software and the Agilent® High Sensitivity DNA Kit	1. Dilute 2 µL of each purified PCR product 1:10 with low TE.<br>2. Analyze 1 µL of each *diluted* purified PCR product with the Agilent® 2100 Bioanalyzer® instrument and an Agilent® High Sensitivity DNA Kit. Use the Agilent® software to determine the amount (ng) of target amplicons.<br>**Note:** See Appendix B, "Example: Calculate DNA input for library preparation".
Quant-iT™ dsDNA Assay Kit, High Sensitivity (Cat. no. Q33120)[1]<br>*or*<br>Qubit™ dsDNA HS Assay Kit (Cat. no. Q32851)	Use 2 µL of each purified PCR product and follow the kit protocol recommendations.

[1] For use with fluorescent plate readers or the Qubit™ 2.0 Fluorometer.

Prepare the library

Use the Ion Plus Fragment Library Kit (Cat. no. 4471252) according to the following procedures. Refer to the *Prepare Amplicon Libraries without Fragmentation Using the Ion Plus Fragment Library Kit User Bulletin* (Pub. no. MAN0006846) for details.

End repair and purify pooled amplicons

1. Combine the following in a 1.5-mL Eppendorf® LoBind® tube:

Component	Volume
Pooled short amplicons, 10–100 ng	79 µL
5X End Repair Buffer	20 µL
End Repair Enzyme	1 µL
**Total**	**100 µL**

2. Pipet up and down to thoroughly mix, then incubate at room temperature for 20 minutes.

3. Add 180 µL Agencourt® AMPure® XP Reagent (1.8 × *sample volume*) to each sample, vortex the mixture briefly, pulse-spin, then incubate for 5 minutes at room temperature.

4. Pulse-spin and place the tube in a magnetic rack such as the DynaMag™-2 magnet for 3 minutes or until the solution is clear. Carefully remove and discard the supernatant without disturbing the pellet.

5. Without removing the tube from the magnet, add 500 µL of 70% ethanol. Incubate for 30 seconds, turning the tube around twice in the magnet to move the beads around. After the solution clears, remove and discard the supernatant without disturbing the pellet.

6. Repeat step 5 for a second wash.

7. To remove residual ethanol, pulse-spin the tube, place it back in the magnetic rack, and carefully remove any remaining supernatant with a 20-µL pipettor without disturbing the pellet.

8. Keeping the tube on the magnetic rack, air-dry the beads at room temperature for 4 minutes.

   IMPORTANT! Do not let the pellet dry out completely.

9. Remove the tube from the magnetic rack and add 25 µL of Low TE directly to the pellet to disperse the beads. Vortex the sample for 5–10 seconds as needed to resuspend the beads.

10. Pulse-spin and place the tube in the magnetic rack for at least 1 minute until the solution clears. Transfer the supernatant containing the eluted DNA to a new 1.5-mL Eppendorf LoBind® Tube without disturbing the pellet.

    IMPORTANT! The supernatant contains the eluted DNA. **Do not discard.**

STOPPING POINT *(Optional)* Store the DNA at –30°C to –10°C.

Ligate and nick-repair

Note: For barcoded libraries, use the Ion Xpress™ Barcode Adapters 1-16 Kit (Cat. no. 4471250) or other similar barcode kits available from **www.lifetechnologies.com** and the following procedure.

1. In a 0.2-mL PCR tube, combine the reagents as indicated in the table, and mix well by pipetting up and down.

Component	Volume for Non-barcoded Libraries	Volume for Barcoded Libraries
DNA	~25 µL	~25 µL
10X Ligase Buffer	10 µL	10 µL
Adapters (non-barcoded libraries)<br>or<br>Ion P1 Adapter (barcoded libraries)	2 µL	2 µL
Ion Xpress™ Barcode X[1]	—	2 µL
dNTP Mix	2 µL	2 µL
Nuclease-free Water	51 µL	49 µL
DNA Ligase	2 µL	2 µL
Nick Repair Polymerase	8 µL	8 µL
**Total**	**100 µL**	**100 µL**

[1] X = barcode chosen.

IMPORTANT! When handling barcoded adapters, be especially careful not to cross-contaminate. Change gloves frequently and open one tube at a time.

Note: Add **both** Ion P1 Adapter and the desired Ion Xpress™ Barcode X adapter to the ligation reaction for barcoded libraries. For non-barcoded libraries, this step is not required.

2. Place the tube in a thermal cycler and run the following program.

Stage	Temperature	Time
Hold	25°C	15 min
Hold	72°C	5 min
Hold	4°C	∞[1]

[1] Not a stopping point; continue directly to the next steps.

3. Transfer the entire reaction mixture to a 1.5-mL Eppendorf LoBind® Tube for the next cleanup step.

Purify the adapter-ligated and nick-repaired DNA

IMPORTANT! Always use 70% ethanol for the next steps. A higher percentage of ethanol causes inefficient washing of smaller-sized molecules. A lower percentage of ethanol may cause sample loss.

1. Add 140 µL (1.4 × *sample volume*) of Agencourt® AMPure® XP Reagent to the sample, vortex the mixture briefly, pulse-spin, then incubate the mixture for 5 minutes at room temperature.

2. Pulse-spin and place the tube in a magnetic rack such as the DynaMag™-2 magnet for 3 minutes or until the solution is clear. Carefully remove and discard the supernatant without disturbing the pellet.

3. Without removing the tube from the magnet, add 500 µL of 70% ethanol. Incubate for 30 seconds, turning the tube around twice in the magnet to move the beads around. After the solution clears, remove and discard the supernatant without disturbing the pellet.

4. Repeat step 3 for a second wash.

5. To remove residual ethanol, pulse-spin the tube, place it back in the magnetic rack, and carefully remove any remaining supernatant with a 20-µL pipettor without disturbing the pellet.

6. Keeping the tube on the magnetic rack, air-dry the beads at room temperature for 4 minutes.

   IMPORTANT! Do not let the pellet dry out completely.

7. Remove the tube from the magnetic rack and add 20 µL of Low TE directly to the pellet to disperse the beads. Vortex the sample for 5–10 seconds as needed to resuspend the beads.

8. Pulse-spin and place the tube in the magnetic rack for at least 1 minute until the solution clears. Transfer the supernatant containing the eluted DNA to a new 1.5-mL Eppendorf® LoBind® Tube without disturbing the pellet.

   IMPORTANT! The supernatant contains the eluted DNA. **Do not discard.**

STOPPING POINT (*Optional*) Store the DNA at –30°C to –10°C.

Determine library concentration

To maximize the number of useful reads from the sequencing run, it is important to determine the optimal library concentration for template preparation. An over-diluted library results in very few reads, while an under-diluted library results in a high proportion of polyclonal beads and decreased number of useful reads.

Use one of the methods below to quantitate libraries in order to determine library concentration.

Quantitation method	Follow this procedure	Recommended library concentration for template preparation
qPCR	"Determine library concentration using qPCR" on page 19	10 pM
Agilent® 2100 Bioanalyzer® instrument	"Determine library concentration using a Bioanalyzer® instrument" on page 22	26 pM

Important guidelines for preparing control and library dilutions:

- We recommend nonstick microfuge tubes (for example, Eppendorf® DNA LoBind® Tubes) for serial dilution preparation.
- Prepare sufficient volume of each dilution for the size of your qPCR reactions and the number of replicates (for example, for a 20-µL qPCR reaction volume, prepare 5 µL of diluted standard per reaction, or 15 µL per triplicate plus a small overage for pipetting loss).

Determine library concentration using qPCR

Use the Ion Universal Library Quantitation Kit (Cat. no. A26217) and the following procedure to quantify libraries prepared using Ion 16S™ Metagenomics Kit amplicons.

Prepare serial dilutions of the *E. coli* DH10B Control Library

1. Thaw the *E. coli* DH10B Ion Control Library on ice. Vortex and briefly spin down before taking aliquots.

2. Prepare four sequential 10-fold dilutions from the *E. coli* DH10B Ion Control Library (68 pM; included in the Ion Universal Library Quantitation Kit) as shown in the table. Vortex and briefly spin down each standard before taking aliquots for the next dilution. Label the standards and store on ice.

Standard	Control Library	Nuclease-free Water[1]	Dilution factor	Concentration
1	5 µL undiluted Control Library	45 µL	1:10	6.8 pM
2	5 µL Std 1	45 µL	1:100	0.68 pM
3	5 µL Std 2	45 µL	1:1000	0.068 pM
4	5 µL Std 3	45 µL	1:10000	0.0068 pM

[1] Not DEPC-treated

Note: When you program the qPCR instrument, enter the concentration of each standard in the "Amount" field.

Dilute the sample library

Prepare dilutions of the sample library that target a concentration within the serial dilutions of the control library, as described in the following table. It is best to prepare 3 independent dilutions for qPCR. At a minimum, prepare 3 technical replicate qPCR reactions of each individual dilution. For a standard 20-µL qPCR reaction, prepare 5 µL of each library dilution per reaction.

Prepare serial dilutions of the sample library as shown in the table. Label the dilutions and store on ice.

Dilution	Library input	Nuclease-free Water[1]
1:10	2 µL of sample library stock	18 µL
1:100	5 µL of 1:10	45 µL
1:1000[2]	5 µL of 1:100	45 µL
1:10,000[2]	5 µL of 1:1000	45 µL

[1] Not DEPC-treated
[2] Dilutions to be assayed.

Set up the PCR reactions

1. Thaw frozen components on ice. Gently but thoroughly mix each thawed component, then briefly centrifuge to bring the contents to the bottom of the tube. Do not vortex the TaqMan® Fast Universal PCR Master Mix.

2. Prepare a reaction mix as shown in the table. Store on ice.

   Note: Scale the volumes based on the number and volume of your qPCR reactions (At a minimum, prepare sufficient volume for three technical replicates of each control dilution and library dilution.)

Component	Volume per 20-µL reaction
TaqMan® Fast Universal PCR Master Mix (2X), No AmpErase® UNG	10 µL
Ion Library TaqMan® Quantitation Assay, 20X	1.0 µL
Nuclease-free Water	4.0 µL

3. Vortex the reaction mix for 5 seconds, pulse-spin, then pipette 15 µL into the appropriate number of wells in the PCR plate.

4. Add 5 µL of the diluted (1:1000 or 1:10000) sample library or 5 µL of Standard 1–Standard 4 to each well (three wells per sample or standard), for a total reaction volume of 20 µL.

   IMPORTANT! Use equivalent volumes of sample library and standards in PCR reactions.

5. Seal the plate, and centrifuge the plate briefly to spin down the contents and eliminate air bubbles.

Run the real-time PCR reactions

The TaqMan® Fast Universal PCR Master Mix (2X), No AmpErase® UNG can be used with the real-time PCR instruments shown in the table.

1. Program your real-time instrument according to the manufacturer's instructions using the following settings.
   - Enter the concentrations of the control library standards (see "Prepare serial dilutions of the E. coli DH10B Control Library" on page 19)
   - Passive reference dye: **ROX™ Reference Dye**
   - TaqMan® probe reporter/quencher: **FAM™ dye/MGB**
   - Reaction volume: **20 µL**
   - Enter the appropriate cycling program shown in the table

Real-time PCR System	Sample block and reaction plate	Run mode	Stage	Temp	Time
7900 HT Fast<br>StepOnePlus™<br>ViiA™ 7	96-well Fast	Fast	Hold	95°C	20 sec
			Cycle (40 cycles)	95°C	1 sec
StepOne™	48-well Fast			60°C	20 sec
7500 Fast	96-well Fast	Fast	Hold	95°C	20 sec
			Cycle (40 cycles)	95°C	3 sec
				60°C	30 sec
7900 HT Fast<br>ViiA™ 7<br>7900 HT	96-well standard<br>384-well	Standard	Hold	95°C	2 min
			Cycle (40 cycles)	95°C	15 sec
7500<br>7300	96-well standard			60°C	1 min

2. Place the plate in the real-time PCR instrument, run the reactions, and collect the real-time data.

Determine the sample library dilution factor using qPCR results

The real-time PCR instrument software calculates the diluted library concentration. Use this information to calculate the *undiluted* library concentration, and the library dilution factor that will yield 10 pM input for template preparation.

1. Calculate the undiluted sample library concentration: Undiluted Library Concentration = (Concentration determined by qPCR) x (Sample Library Dilution (1,000 or 10,000).
   - If both dilutions of the sample library fall within the standard curve, calculate the undiluted library concentration for each dilution, then take the average the two undiluted concentrations
   - Otherwise, calculate the undiluted library concentration of the dilution that falls within the standard curve

   Example: The diluted library concentration determined by qPCR is 5 pM for a 1:1,000 library dilution. Undiluted Library Concentration = (5 pM) x (1,000) = 5,000 pM

2. Determine the library dilution factor. For libraries analyzed using qPCR, dilute the library to a concentration of ~10 pM. This concentration is suitable for downstream template preparation.

   Determine the dilution factor using the following formula:

   Dilution factor = (Undiluted library concentration in pM)/10 pM

   Example:

   The library concentration is 5,000 pM.

   Dilution factor = 5,000 pM/10 pM = 500

   Thus, 2 µL of library mixed with 998 µL of Low TE (1:500 dilution) yields approximately 10 pM.

Use this library dilution for template preparation.

Determine library concentration using a Bioanalyzer® instrument

Amplify the library

Use the components provided with the Ion Plus Fragment Library Kit for this step.

1. Add 5 µL of Low TE to the ~20 µL of purified, adapter-ligated library.

2. Combine the following reagents in an appropriately sized tube and mix by pipetting up and down.

Component	Volume
Platinum™ PCR SuperMix High Fidelity	100 µL
Library Amplification Primer Mix	5 µL
Unamplified library	25 µL
**Total**	**130 µL**

3. Split the 130-µL reaction into two 0.2-mL PCR tubes, each containing about 65 µL.

4. Place the tubes into a thermal cycler and run the following PCR cycling program.

   Note: Minimize the number of cycles to avoid over-amplification, production of concatemers, and introduction of PCR-induced errors. Reduce the number of cycles if concatemers are formed.

Stage	Step	Temperature	Time
Holding	Denature	95°C	5 min
5–7 cycles[1]	Denature	95°C	15 sec
	Anneal	58°C	15 sec
	Extend	70°C	1 min
Holding	—	4°C	Hold for up to 1 hour

[1] 5 cycles for 50 ng of input, 7 cycles for 20 ng of input.

5. Combine previously split PCRs in a new 1.5-mL Eppendorf LoBind® Tube.

Purify the library

IMPORTANT! Always use 70% ethanol for the next steps. A higher percentage of ethanol causes inefficient washing of smaller-sized molecules. A lower percentage of ethanol may cause sample loss.

1. Add 195 µL of Agencourt® AMPure® XP Reagent (1.5 × *sample volume*) to each sample, vortex the mixture briefly, pulse-spin, then incubate the mixture for 5 minutes at room temperature.

2. Pulse-spin and place the tube in a magnetic rack such as the DynaMag™-2 magnet for 3 minutes or until the solution is clear. Carefully remove and discard the supernatant without disturbing the pellet.

3. Without removing the tube from the magnet, add 500 µL of 70% ethanol. Incubate for 30 seconds, turning the tube around twice in the magnet to move the beads around. After the solution clears, remove and discard the supernatant without disturbing the pellet.

4. Repeat step 3 for a second wash.

5. To remove residual ethanol, pulse-spin the tube, place it back in the magnetic rack, and carefully remove any remaining supernatant with a 20-µL pipettor without disturbing the pellet.

6. Keeping the tube on the magnet, air-dry the beads at room temperature for 4 minutes.

7. Remove the tube from the magnetic rack, and add 20 µL of Low TE directly to the pellet to disperse the beads. Vortex the sample for 5–10 seconds as needed to resuspend the beads.

8. Pulse-spin and place the tube in the magnetic rack for at least 1 minute until the solution clears. Transfer the supernatant containing the eluted DNA to a new 1.5-mL Eppendorf LoBind® Tube without disturbing the pellet.

   IMPORTANT! The supernatant contains the final amplified library. **Do not discard.**

STOPPING POINT Store the library at –30°C to –10°C. Before use, thaw on ice. To reduce the number of freeze-thaw cycles, store the library in several aliquots.

Assess the quality of the library

Analyze a 1-µL aliquot of the amplified library, diluted 1:10, on the Bioanalyzer® instrument with an Agilent® High Sensitivity DNA Kit.

IMPORTANT! Ensure that excessive amounts of primer-dimers (immediately adjacent to the marker) or overamplification products (concatemers) are not present. For more information, contact Technical Support.

Determine the sample library dilution factor using the Bioanalyzer® instrument analysis

1. From the Bioanalyzer® instrument analysis used to assess the library size distribution, determine the molar library concentration in pmol/L using the Bioanalyzer® software. If necessary, follow the manufacturer's instructions to perform a region analysis (smear analysis) to place the entire distribution of library molecules within a single peak.

2. Determine the library dilution factor. For libraries analyzed using Bioanalyzer® instrument analysis, dilute the library to a concentration of ~26 pM. This concentration is suitable for downstream template preparation.

   Determine the dilution factor using the following formula:

   Dilution factor = (Library concentration in pM)/26 pM

   Example:

   The library concentration is 10,000 pM.

   Dilution factor = 10,000 pM/26 pM = 385

   Thus, 1 µL of library mixed with 385 µL of Low TE (1:385 dilution) yields approximately 26 pM. Use this library dilution for template preparation.

Proceed to template preparation

Proceed to template preparation, using the Ion OneTouch™ 2 System and the Ion PGM™ Hi-Q™ OT2 Kit. Follow the instructions in the *Ion PGM™ Hi-Q™ OT2 Kit User Guide* (Pub. no. MAN0010902), *with these modifications*:

1. Before you begin template preparation, dilute an aliquot of each library according to the calculations in "Determine the sample library dilution factor using qPCR results" on page 22 or "Determine the sample library dilution factor using the Bioanalyzer® instrument analysis" on page 24, as appropriate.

   Note: If you plan to sequence more than one library on the same chip, dilute each library to 10 pM (if you quantified the library using qPCR), or 26 pM (if you quantified the library using a Bioanalyzer® instrument), then pool the diluted libraries to obtain a final volume ≥25 µL. You can:
   - Pool equal volumes of diluted library
     or
   - Pool volumes in a ratio equivalent to the percentage of wells that you want to use to sequence each library
     For example, if you want to use 45% of the wells for library 1, 45% for library 2, and 10% for library 3, then pool volumes in a 45:45:10 ratio.

   Note: Store diluted libraries at 2°C to 8°C and use within 48 hours. Store undiluted libraries at –30°C to –10°C.

2. Follow the instructions for template preparation as described in Chapter 3 of the *Ion PGM™ Hi-Q™ OT2 Kit User Guide* (Pub. no. MAN0010902) *but do not further dilute the library*. Add 20 µL of diluted library (prepared in step 1) and 5 µL of water to the amplification solution.

3. Remove the samples ≤ 16 hours after starting the run on the Ion OneTouch™ 2 Instrument.

Note: Template preparation documentation is available on the Ion Community at **http://ioncommunity.lifetechnologies.com**. Follow the links under **Protocols ▸ Prepare Template ▸ Prepare Template User Guides and Quick Reference**.

Sequence the library

Use the Ion Personal Genome Machine™ (PGM™) System and the Ion PGM™ Hi-Q™ Sequencing Kit. Follow the protocol in the *Ion PGM™ Hi-Q™ Sequencing Kit User Guide* (Pub. no. MAN0009816).

Use an Ion 314™ Chip v2, Ion 316™ Chip v2, or Ion 318™ Chip v2 depending on the number of barcoded libraries pooled for run, initial sample complexity and/or desired sequencing depth.

Troubleshooting

Observation	Possible cause	Recommended action
PCR product is absent in both samples and positive controls	Errors in reaction set-up.	Repeat, carefully following procedures.
	Insufficient heat tranfer during thermal cycling.	Confirm PCR tube or plate compatibility with your PCR instrument.
PCR product is absent in samples, but present in positive controls	Low amount of microbial DNA in sample.	• Increase volume of input DNA to 12 µL.<br>*or*<br>Increase number of PCR cycles to 30 cycles.<br>• If the amount of microbial DNA is very low, increase both the DNA input and number of PCR cycles.
	PCR inhibitors are present.	• Dilute the sample 1:10 for PCR and use same number of PCR cycles.<br>*or*<br>• Dilute the sample 1:10 for PCR and increase the number of PCR cycles.
PCR product is present in negative control reactions	Negative control (water) is contaminated by previous PCR reactions.	Replace negative control.
	PCR MM and/or PCR primer mixes are contaminated with amplicons from previous PCR reactions.	Include AmpErase® Uracil N-Glycosylase (UNG) Uracil N-Glycosylase (UNG) (Cat. no. N8080096) in the PCR reaction. Follow the instructions in the AmpErase procedures.
Library yield is low as determined by real-time qPCR	The Ion Library Quantitation Kit (Cat. no. 4468802) was used instead of the Ion Universal Library Quantitation Kit.	Use the Ion Universal Library Quantitation Kit (Cat. no. A26217) when using the Ion 16S™ Metagenomics Kit.
	Inefficient adaptor ligation during library preparation due to errors in reaction setup.	Repeat library preparation. Make sure that the components are added in the recommended order.

B Example: Calculate DNA input for library preparation

The required input for the library preparation (end-repair) step is 10–100 ng in 79 µL volume.

Information needed to prepare sample for end-repair step	Value used in example
Target DNA input amount	50 ng
Concentration from Bioanalyzer® instrument traces[1]	2,017.94 pg/µL
Purified PCR product dilution factor	1:10

[1] See Figure 1 .

Purified PCR product concentration (ng/µL):

Concentration from Bioanalyzer® instrument traces × (Dilution Factor/1000)

2,017.94 pg/µL × 10/1000 = 20.18 ng/µL

Volume of purified PCR product needed to obtain 50 ng pooled amplicon:

Target DNA input/purified PCR product concentration

50 ng/20.18 ng/µL = 2.5 µL of DNA (pooled amplicon)

Add 2.5 µL of pooled amplicon to 76.5 µL nuclease-free water to obtain the 50 ng in a 79 µL volume.

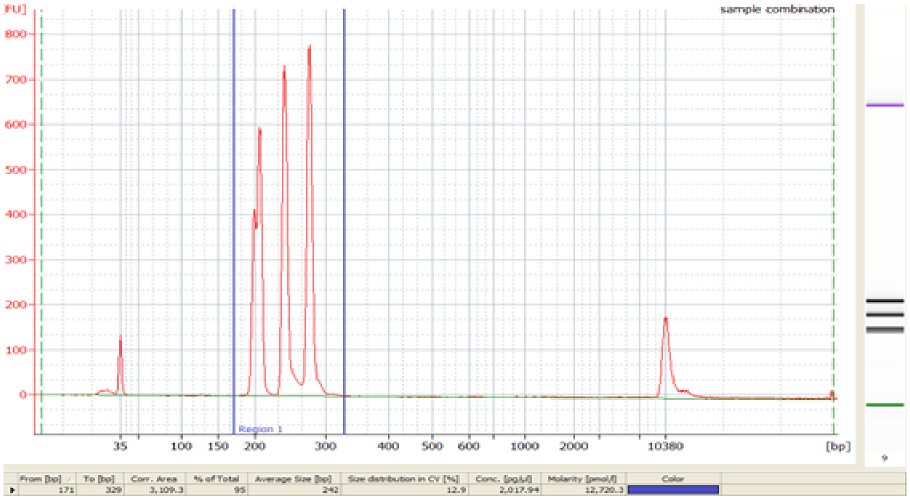

Figure 1 Bioanalyzer® instrument traces for a purified PCR product prepared with the Ion 16S™ Metagenomics Kit

C Good laboratory practices for PCR and RT-PCR

When preparing samples for PCR or RT-PCR amplification:

- Wear clean gloves and a clean lab coat (not previously worn while handling amplified products or during sample preparation).
- Change gloves whenever you suspect that they are contaminated.
- Maintain separate areas and dedicated equipment and supplies for:
  - Sample preparation and reaction setup.
  - Amplification and analysis of products.
- Do not bring amplified products into the reaction setup area.
- Open and close all sample tubes carefully. Avoid splashing or spraying samples.
- Keep reactions and components capped as much as possible.
- Use a positive-displacement pipettor or aerosol-resistant barrier pipette tips.
- Clean lab benches and equipment periodically with 10% bleach solution or DNA*Zap*™ Solutions (Cat. no. AM9890).

D

Safety

WARNING! GENERAL SAFETY. Using this product in a manner not specified in the user documentation may result in personal injury or damage to the instrument or device. Ensure that anyone using this product has received instructions in general safety practices for laboratories and the safety information provided in this document.

- Before using an instrument or device, read and understand the safety information provided in the user documentation provided by the manufacturer of the instrument or device.
- Before handling chemicals, read and understand all applicable Safety Data Sheets (SDSs) and use appropriate personal protective equipment (gloves, gowns, eye protection, etc). To obtain SDSs, see the “Documentation and Support” section in this document.

Chemical safety

WARNING! GENERAL CHEMICAL HANDLING. To minimize hazards, ensure laboratory personnel read and practice the general safety guidelines for chemical usage, storage, and waste provided below, and consult the relevant SDS for specific precautions and instructions:

- Read and understand the Safety Data Sheets (SDSs) provided by the chemical manufacturer before you store, handle, or work with any chemicals or hazardous materials. To obtain SDSs, see the "Documentation and Support" section in this document.
- Minimize contact with chemicals. Wear appropriate personal protective equipment when handling chemicals (for example, safety glasses, gloves, or protective clothing).
- Minimize the inhalation of chemicals. Do not leave chemical containers open. Use only with adequate ventilation (for example, fume hood).
- Check regularly for chemical leaks or spills. If a leak or spill occurs, follow the manufacturer's cleanup procedures as recommended in the SDS.
- Handle chemical wastes in a fume hood.
- Ensure use of primary and secondary waste containers. (A primary waste container holds the immediate waste. A secondary container contains spills or leaks from the primary container. Both containers must be compatible with the waste material and meet federal, state, and local requirements for container storage.)
- After emptying a waste container, seal it with the cap provided.
- Characterize (by analysis if necessary) the waste generated by the particular applications, reagents, and substrates used in your laboratory.
- Ensure that the waste is stored, transferred, transported, and disposed of according to all local, state/provincial, and/or national regulations.
- **IMPORTANT!** Radioactive or biohazardous materials may require special handling, and disposal limitations may apply.

Biological hazard safety

WARNING! BIOHAZARD. Biological samples such as tissues, body fluids, infectious agents, and blood of humans and other animals have the potential to transmit infectious diseases. All work should be conducted in properly equipped facilities using the appropriate safety equipment (for example, physical containment devices). Safety equipment also may include items for personal protection, such as gloves, coats, gowns, shoe covers, boots, respirators, face shields, safety glasses, or goggles. Individuals should be trained according to applicable regulatory and company/ institution requirements before working with potentially biohazardous materials. Follow all applicable local, state/provincial, and/or national regulations. The following references provide general guidelines when handling biological samples in laboratory environment.

- U.S. Department of Health and Human Services, *Biosafety in Microbiological and Biomedical Laboratories (BMBL)*, 5th Edition, HHS Publication No. (CDC) 21-1112, Revised December 2009; found at:
  www.cdc.gov/biosafety/publications/bmbl5/BMBL.pdf
- World Health Organization, *Laboratory Biosafety Manual*, 3rd Edition, WHO/CDS/CSR/LYO/2004.11; found at:
  www.who.int/csr/resources/publications/biosafety/Biosafety7.pdf

Documentation and support

Related documentation

Document	Publication number	Available from
Ion PGM™ Hi-Q™ Sequencing Kit User Guide	MAN0009816	Ion Community at **http://ioncommunity.lifetechnologies.com**. Follow the links under **Protocols ▸ Prepare Template ▸ Prepare Template User Guides and Quick Reference**
Ion PGM™ Hi-Q™ OT2 Kit User Guide	MAN0010902	
(*Optional*) *Prepare Amplicon Libraries without Fragmentation Using the Ion Plus Fragment Library Kit*	MAN0006846	
Agilent® High Sensitivity DNA Kit Guide	G2938-90321	Agilent® Technologies

Note: To open the user documentation, use the Adobe® Reader® software available from **www.adobe.com**

Note: For additional documentation, see "Customer and technical support" on page 32.

Customer and technical support

Visit **thermofisher.com/techresources** for the latest in services and support, including:

- Worldwide contact telephone numbers
- Product support, including:
  - Product FAQs
  - Software, patches, and updates
- Order and web support
- Product documentation, including:
  - User guides, manuals, and protocols
  - Certificates of Analysis
  - Safety Data Sheets (SDSs; also known as MSDSs)

    Note: For SDSs for reagents and chemicals from other manufacturers, contact the manufacturer.

Limited product warranty

Life Technologies Corporation and/or its affiliate(s) warrant their products as set forth in the Life Technologies' General Terms and Conditions of Sale found on Life Technologies' website at **www.lifetechnologies.com/termsandconditions**. If you have any questions, please contact Life Technologies at **www.lifetechnologies.com/support**.

For support visit **thermofisher.com/techresources** or email **techsupport@lifetech.com**
lifetechnologies.com

1 June 2015
